# Decrease of cancer diagnosis during COVID-19 pandemic: a systematic review and meta-analysis

**DOI:** 10.1007/s10654-022-00946-6

**Published:** 2023-01-03

**Authors:** Marco Angelini, Federica Teglia, Laura Astolfi, Giulia Casolari, Paolo Boffetta

**Affiliations:** 1 Department of Medical and Surgical Sciences, University of Bologna, Via Massarenti 9, 40138 Bologna, Italy; 2 Stony Brook Cancer Center, Stony Brook University, New York, NY USA

**Keywords:** COVID-19, Cancer, Cancer diagnosis, Pandemic, Meta-analysis, Diagnostic test, Review

## Abstract

Many health services, including cancer care, have been affected by the COVID-19 epidemic. This study aimed at providing a systematic review of the impact of the epidemic on cancer diagnostic tests and diagnosis worldwide. In our systematic review and meta-analysis, databases such as Pubmed, Proquest and Scopus were searched comprehensively for articles published between January 1st, 2020 and December 12th, 2021. Observational studies and articles that reported data from single clinics and population registries comparing the number of cancer diagnostic tests and/or diagnosis performed before and during the pandemic, were included. Two pairs of independent reviewers extracted data from the selected studies. The weighted average of the percentage variation was calculated and compared between pandemic and pre-pandemic periods. Stratified analysis was performed by geographic area, time interval and study setting. The review was registered on PROSPERO (ID: CRD42022314314). The review comprised 61 articles, whose results referred to the period January–October 2020. We found an overall decrease of − 37.3% for diagnostic tests and − 27.0% for cancer diagnosis during the pandemic. For both outcomes we identified a U-shaped temporal trend, with an almost complete recovery for the number of cancer diagnosis after May 2020. We also analyzed differences by geographic area and screening setting. We provided a summary estimate of the decrease in cancer diagnosis and diagnostic tests, during the first phase of the COVID-19 pandemic. The delay in cancer diagnosis could lead to an increase in the number of avoidable cancer deaths. Further research is needed to assess the impact of the pandemic measures on cancer treatment and mortality.

**Supplementary Information:**

The online version contains supplementary material available at 10.1007/s10654-022-00946-6.

## Introduction

The COVID-19 pandemic caused an extraordinary burden on the healthcare system, patient care and dramatically impacted the delivery of medical services [1].

In order to limit the spread of the infection many governments imposed measures, mostly based on personal distancing (“lockdown”) or declared states of emergency.

Even if needed to control the number of new COVID-19 cases, these interventions negatively affected the diagnosis of other major medical conditions, causing for example, delays in patients with myocardial infarction seeking medical help in China [2], a sustained reduction in the number of people referred and diagnosed for colorectal cancer in the UK [3] and the reduction of HIV testing and diagnosis in many countries in the world [4]. The pandemic measures brought to unprecedented challenges for clinicians to care for oncologic patients as well, as demonstrated by a large study conducted in the US [5]. Cancer care suffered from delayed cancer diagnoses because of reduced cancer screening [6], as well as delays in diagnostic investigations [7, 8] and surgical procedures [9, 10].

Several retrospective studies confirmed a decrease or delay in oncologic surgical procedures, cancer screening tests, clinic visits, and a significant decline in newly identified patients with cancer [5, 11]. As a consequence, delay of the diagnosis may become more frequent, resulting in poorer outcomes [12].

We performed a systematic review and meta-analysis of studies that analyzed the variation in the total number of cancer diagnostic tests and diagnosis since the beginning of the epidemic, in comparison to the previous period.

This article represents the follow-up of a prior systematic review that analyzed the variation in the total number of breast, cervical and colorectal cancer screening tests performed since the beginning of the pandemic [5]. Both articles are part of a research project that aims at quantifying the global impact of COVID-19 measures on cancer burden.

## Materials and methods

### Search strategy and selection criteria

The research protocol, was included in the PROSPERO Register (registration number CRD42022314314) and consisted of a systematic review and meta-analysis conducted according to the PRISMA statement [13]. The study question was developed following the patients, exposure, comparison group, outcomes and study design (PECO) framework [14], where the population under investigation consisted of cancer patients. A comparison between the number of cancer diagnosis and diagnostic tests occurred in a previous period and during the COVID-19 pandemic was made, in order to obtain their percentage variation as outcome.

This study is part of a larger project that aims to assess the global impact of the COVID-19 pandemic on cancer patients, including not only cancer diagnosis and diagnostic tests, but also cancer screening, treatments and medical visits for oncologic patients. The search string was unique for all these outcomes and was launched on PubMed, Proquest and Scopus, without language restriction, for studies published between January 1, 2020, and December 12, 2021, when the search strings, available in the Supplementary Table 1, were launched. The search string included Boolean operators (AND, OR), Mesh terms and their builder options as relevant, to improve the search results. Searches were performed employing the following terms: *neoplasms, diagnosis, drug therapy, radiotherapy, surgery, therapy, diagnosis, epidemiology, prevention and control, early detection of cancer, COVID-19, organization and administration.*

Observational studies and articles reporting data from cancer registries were selected if specified the periods before and after the beginning of COVID-19 pandemic, with results indicated as number of diagnosis or diagnostic tests performed, or the percentage variation between the two time periods.

We considered eligible the studies presenting the number of cancer diagnosis and/or diagnostic tests performed in 2 or more different time intervals, whose at least one before and one during the COVID-19 pandemic. Articles reporting a percentage variation, in alternative or in addition to the absolute number of the events considered, were included. Studies not reporting a clear indication of the periods of observation were excluded, as well as the ones not reporting data after the beginning of the pandemic.

If the comparison was made between two or more pre-COVID-19 periods, we selected as reference the one closest to the COVID-19 epidemic (for example we considered 2019 if data for both 2019 and 2018 were available).

### Data collection and quality assessment

**Fig. 1:**
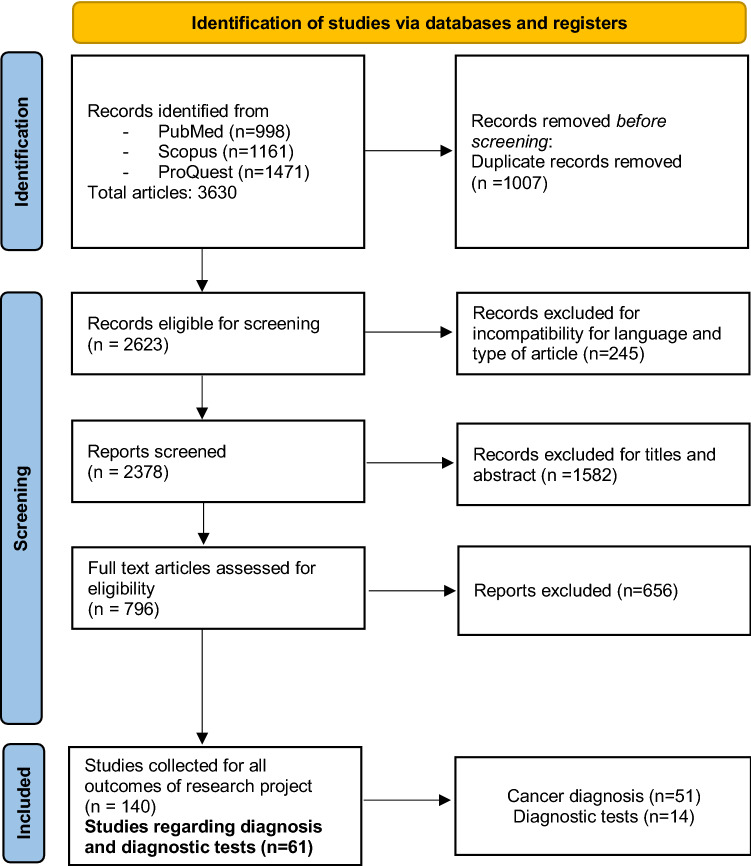
PRISMA (Preferred reporting items for systematic reviews and meta-analyses) flow diagram

Figure 1 illustrates the PRISMA flow diagram of the processes of identification, screening and inclusion of the articles in our systematic review and meta-analysis. We identified a total of 3630 articles: 998 articles on Pubmed, 1471 on Proquest and 1161 on Scopus. We collected initial references in citation files, removed duplicates (1007 articles) and started the screening process of the remaining 2623 articles. We excluded articles in languages other than English, French, Spanish or Italian, as well as reviews, meta-analysis or case reports (a total of 245 articles). After this process, articles were first reviewed by title and then by abstract, resulting in the exclusion of 1582 non-relevant articles. Finally, we examined the full text of each selected article against eligibility criteria and retained 140 studies for all the primary outcomes of the research project (screening, diagnosis, diagnostic tests, visits and therapies), in particular we retained 51 and 14 articles for cancer diagnosis and diagnostic tests, respectively, including 4 articles which reported results for both outcomes.

The title and abstract of the articles were independently reviewed by 2 pairs of reviewers (FT and GC; MA and LA) for inclusion in the first screening phase, followed by the full-text selection phase against eligibility criteria. Disagreements among reviewers in the initial abstract screening phase and full-text review were resolved through discussion with a fifth reviewer (PB).

Two pairs of reviewers (FT and GC; MA and LA) went through a predefined checklist to extract the following information: name of the first author, year of publication, country and type of setting (hospitals and private centers were considered as clinic-based setting, local and national cancer registries were considered as population based), site of cancer, type of diagnostic test performed (pathologic-histologic or other) and absolute number of diagnosis and/or diagnostic tests performed or percentage variation in the number of events between time intervals before and after the beginning of the pandemic.

We also performed a quality assessment of all the studies included in our review using 9 on the 10 items of the Critical Appraisal Skills Programme (CASP) score for qualitative research [15], with a maximum of 10 points. Studies obtaining less than 7 points were considered inadequate and excluded from the meta-analysis (no article was excluded due to low quality score).

Supplementary tables 2 and 3 list the articles included in the present analysis, their major characteristics and quality assessment.

### Statistical analyses

The methods of the statistical analysis were the same as for the first article on this research project [5]. For each outcome, we calculated the weighted average of the percent variation between periods before and after the beginning of COVID-19 pandemic. The weight was calculated using the natural logarithm of the number of daily events in the pre-pandemic period (*daily_events_precovid* obtained dividing number of diagnosis or diagnostic tests in the pre-pandemic period by its duration in days).

$${\text{weight}} = \left| {\ln \left( {{\text{daily}}\_{\text{events}}\_{\text{precovid}}} \right)} \right|$$

We used the logarithm because of the great variability in the number of tests between studies, and selected the absolute value in order to avoid negative weights.

Some studies did not provide the absolute number of cancer diagnosis or diagnostic tests performed, but only the measure of variation between the period on exam and the period used as reference. For these studies, in order to calculate their weight, we imputed a value of *daily_events_precovid*, as mean value of this variable in the studies having the same type of setting and outcome.

We divided the pandemic period into five time intervals, based on the beginning date of the COVID-19 period examined in each article as follows: Period 1 from January 1, 2020 to February 29, 2020; Period 2 from March 1, 2020 to March 31, 2020; Period 3 from April 1, 2020 to April 30 2020, Period 4 from May 1, 2020 to May 31, 2020; Period 5 from June 1, 2020 to October 31, 2020. We did not include in this meta-analysis studies in which the pandemic period started before January 2020. We did not find studies in which the period of observation started after October 2020.

Since each period analyzed is determined only by the starting date of the observation, for some studies it could include an interval longer than our predefined periods (e.g., an observation that considered January 1, 2020 as the starting date and May 31, 2020 as the end date, was only included in the first period).

Studies could contribute to more than one period, but not more than once to each period. In order not to count multiple times studies reporting data for the same outcome or for the same period, we calculated the mean weighted variation of all tumors or all the time intervals reported in each single study, if not already present, obtaining a representative value for the referred outcome.

For example, if an article reported the percentage variation of the diagnosis for 3 different cancers, we calculated the weighted average of the 3 values and used it in assessing the total variation when all cancers were considered together (for example to assess the variation of the total cancer diagnosis in a specific period or geographic area). The same strategy was used in case of data from different periods for the same cancer and outcome. Giving another example, if an article reported the variation in the number of diagnostic liver biopsies along 4 different periods, a weighted mean value was used when analyzing the overall variation along all the pandemic periods.

Additional analyses were performed for specific geographic areas and for type of setting.

We finally fitted multivariate linear models with percentage change as dependent variables for type of structure, geographic area and period. Since a percentage reduction could not be lower than − 100%, we limited the values of the confidence intervals to − 100.0% in case they exceeded it.

We considered the funnel plot and performed the Egger’s regression asymmetry test to assess publication bias [16].

No ethics committee approval was necessary because the study was restricted to publicly available data. For all statistical analyses, we used STATA version 16.1 (Stata Corp., College Station, TX, US).

This research was supported by internal resources of the participating institutions.

## Results

We retained 51 articles for cancer diagnosis and 14 for diagnostic tests, including 4 of which reported results for both outcomes, as showed in the PRISMA flow diagram (Fig. 1). None of these articles was excluded due to low quality score.

### Diagnostic tests

The overall variation in the number of total diagnostic tests for cancer performed throughout the period January-October 2020, compared to the pre-COVID-19 period, was equal to − 37.3% (95% CI: − 44.9; − 29.7, global weighted variation) and specifically, pathologic-histologic tests decreased by − 32.3% (95%CI: − 43.3; − 21.3). The weighted average variation for studies with clinic-based setting experienced a more pronounced decrease − 52.9% (95%CI: − 76.6; − 29.1), than the population-based ones − 35.6% (95% CI: − 44.1; − 27.1). (Table 1).

**Table 1:** Weighted variation of cancer diagnostic tests stratified by period, study setting, geographic area and type of cancer

	Weighted variation	95% CI
*Period*		
January–February March April May June–October	− 20.6% − 40.9% − 53.6% − 51.8% − 32.8%	− 90.1; 48.9 − 49.2; − 32.7 − 79.2; − 28.0 − 70.6; − 33.1 − 48.0; − 17.7
*Study setting*		
Clinic-based Population-based	− 52.9% − 35.6%	− 76.6; − 29.1 − 44.1; − 27.1
*Geographic area*		
North America	− 32.2%	− 55.2; − 9.2
Europe	− 52.6%	− 96.4; − 8.7
South America	− 39.7%	− 50.8; − 28.6
*Cancer site*		
Breast Colorectum	− 41.8% − 47.2%	− 80.4; − 3.2 − 78.2; − 16.3

*CI* Confidence interval

**Fig. 2:**
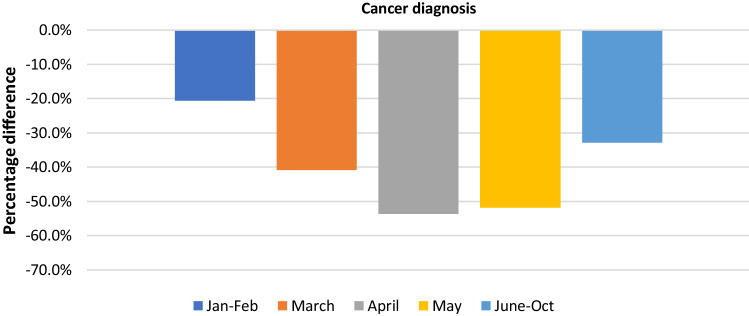
Weighted average variation of cancer diagnostic tests from January to October 2020 compared to pre pandemic period, divided by time period.

The temporal trend displayed the maximum variation in April 2020 (− 53.6%, 95%CI: − 79.2; − 28.0) and a significant reduction was still present from May onwards (Fig. 2 and Table 1).

We also analyzed the 3 main geographic areas, whose distribution is reported in Supplementary Fig. 1. The weighted average variation, along the entire COVID-19 period was: − 32.2% for North America, − 52.6%, for Europe and − 39.7% for South America (Table 1).

In our linear regression analysis, there was no statistically significant difference for this outcome between geographic areas, periods (using January–February as reference) or types of setting (Table 2).

**Table 2:** Decrease (%) in cancer diagnostic tests – results of multivariate analysis

	Coefficient	95% CI
*Period*		
January–February	Ref	
March	− 18.4	− 53.3; 16.5
April	− 28.4	− 64.5; 7.6
May	− 27.6	− 63.2; 7.9
June–October	− 9.6	− 45.3; 26.1
*Geographic area*		
North America	Ref	
Europe	0.0	− 32.7; 32.7
South America	8.1	− 5.8; 22.1
*Setting of studies*		
Clinic based	Ref	
Population based	11.6	− 12.0; 35.3

*CI* Confidence interval, *Ref* Reference category

The stratification for cancer site was performed only for breast and colorectal cancer, respectively − 41.8%, (95%CI: − 80.4, − 3.2) and − 47.2% (95%CI: − 78.2, − 16.3), due to the small number of observations available for the other cancer groups.

There was no evidence of publication bias, either based on funnel-plot asymmetry or according to Egger regression test, *P* values: 0.74 for diagnosis, 0.95 for diagnostic tests.

### Cancer diagnosis

**Fig. 3:**
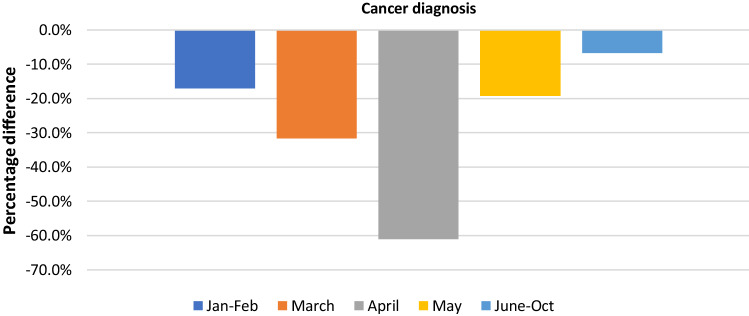
Weighted average variation of cancer diagnosis from January to October 2020 compared to pre-pandemic period, divided by time period.

The average variation of new cancer diagnosis throughout January-October 2020 was − 27.0% (95% CI: − 32.2, − 21.8) compared to the pre-COVID-19 period. In particular, the maximum decline was in April 2020 (− 61.0%, 95% CI: − 76.2; − 45.7), while from May onwards we did not observe a significant variation (Fig. 3 and Table 3).

**Table 3:** Weighted mean variation of cancer diagnosis stratified by period, study setting, geographic area and type of cancer

	Weighted mean variation	95% CI
*Period*		
January–February March April May June–October	− 17.0% − 31.7% − 61.0% − 19.3% − 6.7%	− 25.2; − 8.8 − 37.9; − 25.5 − 76.2, − 45.7 − 51.7; 13.1 − 16.4; 3.1
*Study setting*		
Clinic-based Population-based	− 27.7% − 26.8%	− 37.7; − 17.6 − 33.1; − 20.4
*Geographic area*		
North America	− 27.5%	− 36.2; − 18.8
Asia	− 29.6%	− 52.6; − 6.5
Europe	− 25.4%	− 33.6; − 17.3
*Type of cancer*		
Breast	− 19.8%	− 29.3; − 10.2
Genito-urinary Prostate Gastro-intestinal Colorectum Skin Melanoma	− 28.9% − 26.2% − 25.1% − 24.1% − 27.6% − 22.6%	− 37.9; − 20.0 − 37.6; − 14.8 − 32.0; − 18.2 − 34.2; − 13.9 − 37.9; − 17.4 − 36.4; − 8.9

*CI* confidence interval

The three main geographic areas showed a significant variation, similar for North America, Asia and Europe: − 27.5%, − 29.6% and − 25.4% respectively (Table 3). The distribution of the data by geographic area is showed in the Supplementary Fig. 2.

Stratifying by study setting, clinic-based and population-based studies showed a comparable reduction: − 27.7% (95%CI: − 37.7; − 17.6) and − 26.8% (95%CI: − 33.1; − 20.4), respectively (Table 3).

The mean decrease in cancer diagnoses varied by tumor site. The minimum variation was observed for breast cancer (− 19.8%, 95%CI: − 29.3; − 10.2) and the maximum for genito-urinary tumors (− 28.9%, 95%CI: − 37.9, − 20.0). Gastro-intestinal (GI) tumors showed a mean reduction of − 25.1% (95% CI: − 32.0; − 18.2), and skin cancer of − 27.6% (95% CI: − 37.9; − 17.2).

In the linear regression model, a statistically significant larger decrease was found for March, April and May compared to January–February, for Europe compared to North America and for GI and skin cancer, compared to breast cancer (Table 4).

**Table 4:** Decrease (%) in cancer diagnosis – results of multivariate analysis

	Coefficient	95% CI
*Period*		
January–February	Ref	
March	− 25.7	− 37.8; − 13.5
April	− 67.6	− 85.2; − 50.0
May	− 35.6	− 53.9; − 17.4
June–October	0.6	− 11.9; 13.2
*Geographic area*		
North America	Ref	
Europe	− 14.4	− 24.4; − 4.4
Asia	− 14.5	− 36.7; 7.7
*Setting of studies*		
Clinic-based	Ref	
Population-based	− 11.6	− 23.3; 0.0
*Cancer*		
Breast Gastro-intestinal Genito-urinary Skin	Ref − 21.8 − 1.2 − 22.5	− 35.0; − 8.5 − 19.5; 17.2 − 34.5; − 10.5

*CI* Confidence interval, *Ref* Reference category

## Discussion

The restrictions imposed to fight the COVID-19 epidemic led to a postponement of many medical services [17, 18], including cancer screening [5] and diagnosis. Consequently, there would be a reduction in cancer diagnostic tests and diagnosis performed during the pandemic, that our research quantified to be − 37.3% and − 27.0% respectively.

Both cancer diagnosis and diagnostic tests decreased, during the pandemic, following a U-shaped trend, with a negative peak in April 2020, when most of the countries in the world applied restrictive measures to avoid the spread of the disease, and an almost complete recover to the pre-pandemic level for diagnosis but not for diagnostic tests in the last period we analyzed (June–October 2020).

A remarkable difference was found between diagnostic tests performed in clinic-based settings (− 52.9%) and population-based ones (− 35.6%); on the other hand, this divergence was not found for cancer diagnosis. This discordance could be explicated underlining that clinic-based studies only consider the decrease of patients attending the hospital or center, while population-based studies analyze the general population, including also subjects who didn’t receive any diagnostic test or diagnosis in the pre-pandemic period.

The greater decrease in diagnostic tests could be explained knowing that those are mostly performed after a positive cancer screening test or during the clinic assessment of patients. Indeed, the effect of the suspension of these services and the “stay at home” orders, could have been to increase the positive predictive value of the diagnostic tests, selecting the patients that needed to be tested urgently. In fact, the more pronounced reduction in diagnostic tests than diagnosis, is also present when stratifying for geographic area (in Europe − 52.6% vs − 25.4% and in North America − 32.2% vs − 27.5%, respectively) and for cancer site (breast cancer − 41.8% vs − 19.8% and colorectal cancer − 47.2% vs − 24.1%). In particular, Europe showed the greatest decrease in diagnostic tests (Table 1). Similarly, in our previous analysis of cancer screening, we found that the decrease in colorectal and breast cancer screening tests was larger in Europe, compared to other geographic areas [5], possibly contributing to the results observed in this analysis.

Besides underdiagnosis [19], the probable consequence of the suspension of cancer care service, together with the lengthening of the time interval from referral to diagnosis [20], is a shift to higher-stage tumors. In the acute phase of the pandemic, the “lockdown” measures and the people’s fear of the infection, brought to a selection of the patients presenting at the hospitals, which were more likely to be the ones with serious concerns for their health. In fact, several studies reveal, besides a decrease in the number of diagnoses, a greater proportion of later stage cancers during 2020 [21, 22]. In Supplementary Table 4 we provided a synthesis of the articles reporting the differences of cancer’s characteristics at diagnosis in the pre-COVID-19 period and in the pandemic one.

On the other hand, the restart of the cancer screening and care services probably caused a rebound of the incidence, with a delayed diagnosis of the cases that were missed during the acute phase of the pandemic [23]. In this scenario, it is reasonable to expect in the next period an increase of cancer incidence and possibly a quantitative shift to advanced-stage cancers. This issue will mainly concern for the types of cancer with faster growth, in which a later stage at diagnosis could importantly influence the type of treatment and its effectiveness. These phenomena are expected to result in an increase of avoidable cancer-related deaths [24, 25].

The phenomenon of aggressive risk-aversion, through public announcements that urged people to stay at home at almost any cost, is probably one of the most important causes of the outcomes outlined in our analysis. Studies published elsewhere (20, 26) suggested that this behavior can harm people and that educational media campaigns to encourage people to seek help when needed, are vital to prevent patients late presentation to a GP.

Our review suffers from some limitations. Firstly, considerable heterogeneity between countries is present in terms of service’s accessibility and participation of the target population, lockdown measures, incidence of COVID-19 and its temporal trend; all of these factors cannot be considered in our statistical analysis because of the small number of studies conducted in each country. Moreover, the attribution of an observation to one of the five periods is based on the beginning date of the time interval; this might lead to non-differential misclassification, resulting in an underestimation of the differences between periods. As mentioned in the Methods, we had to estimate the number of daily events for those studies which did not present this information, in order to calculate their weight.

On the other hand, the generalizability of our results is increased by the large number of studies included, whose data refer to 27 countries all over the world, by the analysis of the outcomes on different cancer sites and by the inclusion of the great majority of the articles that are supposed to be published on the this topic, as shown in Supplementary Fig. 3 and 4.

In summary, our research found that oncologic diagnostic tests decreased more than cancer diagnosis. We observed an U-shaped temporal trend with the greatest decrease in April 2020, for both outcomes. Clinic-based studies showed a larger decrease than the population-based ones.

Future studies on the trends of cancer incidence and mortality, will need to be performed in order to clarify long-term implications and to adopt adequate public health strategies.

## Supplementary Information

Below is the link to the electronic supplementary material.

Supplementary file1 (DOCX 306 KB)

## Data Availability

Tables including the main information about all the studies considered in the article (title, authors, year of publication, country, compared periods, setting, quality assessment score) are available in the supplementary material. The study protocol and the search terms for our research are available in the supplementary material. No unpublished material or study has been used in our article. No informed consent was necessary since no personal information is has been used in our article.
