# Supplementary material for: Decrease of cancer diagnosis during COVID-19 pandemic: a systematic review and meta-analysis
Source: Eur J Epidemiol. 2023 Jan 3;38(1):31–8. doi: 10.1007/s10654-022-00946-6 (PMC9807424; doi:10.1007/s10654-022-00946-6)
Supplement: Supplementary file 1 — Supplementary file1 (DOCX 306 KB) [file 10654_2022_946_MOESM1_ESM.docx]

**Supplementary Materials**

**Supplementary Table 1** Search strings for systematic searches of the published literature

**Supplementary Table 2** Characteristics of studies selected for cancer diagnosis

**Supplementary Table 3** Characteristics of studies selected for cancer diagnostic tests

**Supplementary Table 4** Selected studies on differences of cancer’s characteristics at diagnosis in the pre-COVID-19 (reference period) and COVID-19 pandemic (study period)

**Supplementary Figure 1** Representation of geographic areas of the studies included in the analysis for cancer diagnostic test

**Supplementary Figure 2** Representation of geographic areas of the studies included in the analysis for cancer diagnosis

**Supplementary Figure 3** Representation of the online publication date of the studies included in the analysis for cancer diagnostic test by quarter

**Supplementary Figure 4** Representation of the online publication date of the studies included in the analysis for cancer diagnosis by quarter

**Supplementary Materials**

**Supplementary Table 1: Search strings for systematic searches of the published literature**

**PUBMED**

#1 ((( "Neoplasms/diagnosis"[Mesh] OR "Neoplasms/epidemiology"[Mesh] OR "Neoplasms/prevention and control"[Mesh] )) OR "Early Detection of Cancer"[Mesh]) AND ( "COVID-19/epidemiology"[Mesh] OR "COVID-19/organization and administration"[Mesh] )

#2 ( "Neoplasms/drug therapy"[Mesh] OR "Neoplasms/radiotherapy"[Mesh] OR "Neoplasms/surgery"[Mesh] OR "Neoplasms/therapeutic use"[Mesh] OR "Neoplasms/therapy"[Mesh] ) AND ("COVID-19/organization and administration"[Mesh] OR "COVID-19/epidemiology"[Mesh])

#3 #1 OR #2

#4 #3 AND published from January 1, 2020 to December 12, 2021

**PROQUEST**

#1 ((MESH.EXACT("Neoplasms -- diagnosis") OR MESH.EXACT("Neoplasms -- epidemiology") OR MESH.EXACT("Neoplasms -- prevention & control") OR MESH.EXACT("Early Detection of Cancer"))

#2 ((MESH.EXACT("COVID-19")) OR ((MESH.EXACT("Neoplasms -- surgery") OR MESH.EXACT("Neoplasms -- therapy") OR MESH.EXACT("Neoplasms -- radiotherapy") OR MESH.EXACT("Neoplasms -- drug therapy")) AND MESH.EXACT("COVID-19"))

#3 #1 OR #2

#4 #3 AND published from January 1, 2020 to December 12, 2021

**SCOPUS**

#1 ( KEY ( ( neoplasms  AND  diagnosis )  OR  ( neoplasms  AND  prevention  AND  control )  OR 
( early  AND  detection  AND  of  AND  cancer ) )  AND  ( covid-19  AND  epidemiology ) )

#2 ( KEY ( ( neoplasm  AND therapy )  OR  ( neoplasm  AND surgery )  OR  ( neoplasm  AND 
radiotherapy )  OR  ( neoplasm  AND drug  AND therapy )  OR  ( neoplasm  AND therapeutic  AND use ) )  AND  ( covid-19  AND epidemiology ) )

#3 #1 OR #2

#4 #3 AND published from January 1, 2020 to December 12, 2021

**Supplementary Table 2 Characteristics of studies selected for cancer diagnosis**

| **Reference** | **Country** | **Contrast period** | **Period of study** | **Site of cancer** | **Setting and source of data** | **Quality score** |
| --- | --- | --- | --- | --- | --- | --- |
| (1) | Netherlands | January 7, 2019 – April 28, 2019 | January 6, 2020 – April 26, 2020 | Breast | Netherlands Cancer Registry | 7.5 |
| (2) | Turkey | March 15, 2019 – June 1, 2019 | March 15, 2020 – June 1, 2020 | Miscellaneous | Ankara Oncology Training and Research Hospital | 10 |
|  | Turkey | March 15, 2019 – June 1, 2019 | March 15, 2020 – June 1, 2020 | Miscellaneous | Ankara City Hospital, Ankara Diskapi Yildirim Beyazit Training and Research Hospital, and Gulhane Training and Research Hospital |  |
| (3) | Italy | March 3, 2017 – May 3, 2017 | March 3, 2020 – May 3, 2020 | Solid cancer | Pediatric Oncology Unit of the Istituto Nazionale Tumori, Milan | 7.5 |
|  | Italy | March 3, 2018 – May 3, 2018 | March 3, 2020 – May 3, 2020 | Solid cancer | Pediatric Oncology Unit of the Istituto Nazionale Tumori, Milan | 7.5 |
|  | Italy | March 3, 2019 – May 3, 2019 | March 3, 2020 – May 3, 2020 | Solid cancer | Pediatric Oncology Unit of the Istituto Nazionale Tumori, Milan | 7.5 |
| (4) | USA | March 18, 2019 – April 24, 2019 | March 18, 2020 – April 24, 2020 | Prostate | EHR data from Vanderbilt University Medical Center’s (VUMC) Research and Synthetic Derivatives | 9.5 |
| (5) | Italy | February 22, 2018 – May 3, 2018 | February 22, 2020 – May 3, 2020 | Melanoma | Fondazione IRCCS Policlinico San Matteo | 9 |
|  | Italy | February 22, 2019 – May 3, 2019 | February 22, 2020 – May 3, 2020 | Melanoma | Fondazione IRCCS Policlinico San Matteo | 9 |
| (6) | Faroe Islands | January 1, 2016 – December 31, 2019 | January 1, 2020 – January 31, 2020 | Miscellaneous | The Faroese Cancer Registry (FCR) | 9.5 |
|  | Faroe Islands | January 1, 2016 – December 31, 2019 | February 1, 2020 – Febraury 29, 2020 | Miscellaneous | The Faroese Cancer Registry (FCR) | 9.5 |
|  | Faroe Islands | January 1, 2016 – December 31, 2019 | March 1, 2020 – March 31, 2020 | Miscellaneous | The Faroese Cancer Registry (FCR) | 9.5 |
|  | Faroe Islands | January 1, 2016 – December 31, 2019 | April 1, 2020 – April 30, 2020 | Miscellaneous | The Faroese Cancer Registry (FCR) | 9.5 |
|  | Faroe Islands | January 1, 2016 – December 31, 2019 | May 1, 2020 – May 31, 2020 | Miscellaneous | the Faroese Cancer Registry (FCR) | 9.5 |
|  | Faroe Islands | January 1, 2016 – December 31, 2019 | June 1, 2020 – June 30, 2020 | Miscellaneous | The Faroese Cancer Registry (FCR) | 9.5 |
|  | Faroe Islands | January 1, 2016 – December 31, 2019 | July 1, 2020 – July 31, 2020 | Miscellaneous | The Faroese Cancer Registry (FCR) | 9.5 |
|  | Faroe Islands | January 1, 2016 – December 31, 2019 | August 1, 2020 – August 31, 2020 | Miscellaneous | the Faroese Cancer Registry (FCR) | 9.5 |
|  | Faroe Islands | January 1, 2016 – December 31, 2019 | September 1, 2020 – September 30, 2020 | Miscellaneous | The Faroese Cancer Registry (FCR) | 9.5 |
|  | Faroe Islands | January 1, 2016 – December 31, 2019 | October 1, 2020 – October 31, 2020 | Miscellaneous | The Faroese Cancer Registry (FCR) | 9.5 |
|  | Faroe Islands | January 1, 2016 – December 31, 2019 | November 1, 2020 – November 30, 2020 | Miscellaneous | The Faroese Cancer Registry (FCR) | 9.5 |
|  | Faroe Islands | January 1, 2016 – December 31, 2019 | December 1, 2020 – December 31, 2020 | Miscellaneous | The Faroese Cancer Registry (FCR) | 9.5 |
| (7) | Brazil | January 1, 2019 – December 31, 2019 | January 1, 2020 – August 31, 2020 | Miscellaneous | Public database of SUS (DATASUS) | 9.5 |
| (8) | Italy | March 11, 2019 – May 19, 2019 | March 9, 2020 – May 17, 2020 | Miscellaneous | Secondary Care Hospital Network in the Province of Macerata | 9.5 |
|  | Italy | March 11, 2019 – May 19, 2019 | March 9, 2020 – May 17, 2020 | Prostate | Secondary Care Hospital Network in the Province of Macerata | 9.5 |
|  | Italy | March 11, 2019 – May 19, 2019 | March 9, 2020 – May 17, 2020 | Bladder | Secondary Care Hospital Network in the Province of Macerata | 9.5 |
|  | Italy | March 11, 2019 – May 19, 2019 | March 9, 2020 – May 17, 2020 | Colorectal | Secondary Care Hospital Network in the Province of Macerata | 9.5 |
|  | Italy | March 11, 2019 – May 19, 2019 | March 9, 2020 – May 17, 2020 | Hematologic cancer | Secondary Care Hospital Network in the Province of Macerata | 9.5 |
|  | Italy | March 11, 2019 – May 19, 2019 | March 9, 2020 – May 17, 2020 | Breast | Secondary Care Hospital Network in the Province of Macerata | 9.5 |
|  | Italy | March 11, 2019 – May 19, 2019 | March 9, 2020 – May 17, 2020 | Liver | Secondary Care Hospital Network in the Province of Macerata | 9.5 |
|  | Italy | March 11, 2019 – May 19, 2019 | March 9, 2020 – May 17, 2020 | Stomach | Secondary Care Hospital Network in the Province of Macerata | 9.5 |
|  | Italy | March 11, 2019 – May 19, 2019 | March 9, 2020 – May 17, 2020 | Lung | Secondary Care Hospital Network in the Province of Macerata | 9.5 |
|  | Italy | March 11, 2019 – May 19, 2019 | March 9, 2020 – May 17, 2020 | Melanoma | Secondary Care Hospital Network in the Province of Macerata | 9.5 |
|  | Italy | March 11, 2019 – May 19, 2019 | March  9, 2020 – May 17, 2020 | Pancreas | Secondary Care Hospital Network in the Province of Macerata | 9.5 |
| (9) | USA, Massachusetts | March 2, 2019 – June 2, 2019 | March 2, 2020 – June 2, 2020 | Miscellaneous | Massachusetts General Brigham | 9.5 |
|  |  |  |  |  |  |  |
| (10) | Italy | January 1, 20019 – October 31, 2019 | January 1, 2020 – October 31, 2020 | Colorectal | Survey from 49 GE services affiliated to AIGO, SIED and SIGE | 9.5 |
|  | Italy | January 1, 2019 – October 31, 2019 | January 1, 2020 – October 31, 2020 | Pancreas | Survey from 49 GE services affiliated to AIGO, SIED and SIGE | 9.5 |
| (11) | USA | February 1, 2019 – February 28, 2019 | February 1, 2020 – February 29, 2020 | Breast | Breast Cancer Surveillance Consortium | 10 |
|  | USA | March 1, 2019 – March 31, 2019 | March 1, 2020 – March 31, 2020 | Breast | Breast Cancer Surveillance Consortium | 10 |
|  | USA | April 1, 2019 – April 30, 2019 | April 1, 2020 – April 30, 2020 | Breast | Breast Cancer Surveillance Consortium | 10 |
|  | USA | May 1, 2019 – May 31, 2019 | May 1, 2020 – May 31, 2020 | Breast | Breast Cancer Surveillance Consortium | 10 |
|  | USA | June 1, 2019 – June 30, 2019 | June 1, 2020 – June 30, 2020 | Breast | Breast Cancer Surveillance Consortium | 10 |
|  | USA | July 1, 2019 – July 31, 2019 | July 1, 2020 – July 31, 2020 | Breast | Breast Cancer Surveillance Consortium | 10 |
| (12) | USA | January 1, 2019 - February 28, 2020 | March 1, 2020 – May 31, 2020 | Prostate | International Statistical Classification of Diseases, Tenth Revision, Clinical Modification (ICD-10-CM) | 10 |
|  | USA | January 1, 2019 - February 28, 2020 | March 1, 2020 – May 31, 2020 | Breast | International Statistical Classification of Diseases, Tenth Revision, Clinical Modification (ICD-10-CM) | 10 |
|  | USA | January 1, 2019 - February 28, 2020 | March 1, 2020 – May 31, 2020 | Colorectal | International Statistical Classification of Diseases, Tenth Revision, Clinical Modification (ICD-10-CM) | 10 |
|  | USA | January 1, 2019 - February 28, 2020 | March 1, 2020 – May 31, 2020 | Lung | International Statistical Classification of Diseases, Tenth Revision, Clinical Modification (ICD-10-CM) | 10 |
|  | USA | January 1, 2019 - February 28, 2020 | March 1, 2020 – May 31, 2020 | Pancreas | International Statistical Classification of Diseases, Tenth Revision, Clinical Modification (ICD-10-CM) | 10 |
|  | USA | January 1, 2019 - February 28, 2020 | March 1, 2020 – May 31, 2020 | Cervix | International Statistical Classification of Diseases, Tenth Revision, Clinical Modification (ICD-10-CM) | 10 |
|  | USA | January 1, 2019 - February 28, 2020 | March 1, 2020 – May 31, 2020 | Stomach | International Statistical Classification of Diseases, Tenth Revision, Clinical Modification (ICD-10-CM) | 10 |
|  | USA | January 1, 2019 - February 28, 2020 | March 1, 2020 – May 31, 2020 | Esophageal | International Statistical Classification of Diseases, Tenth Revision, Clinical Modification (ICD-10-CM) | 10 |
| (13) | Italy | March 1, 2019 – December 31, 2019 | March 1, 2020 – December 31, 2020 | Head and neck | Department of Oral and Maxillofacial Sciences. Sapienza University of  Rome and Umberto I of Rome | 8 |
| (14) | Croatia | January 1, 2019 – December 31, 2019 | January 1, 2020 – December 31, 2020 | Breast | 25 Croatian hospitals | 9.5 |
|  | Croatia | January 1, 2019 – December 31, 2019 | June 1, 2020 – December 31, 2020 | Breast | 25 Croatian hospitals | 9.5 |
| (15) | Poland | March 14, 2019 – May 25, 2019 | March 14, 2020 – May 25, 2020 | Miscellaneous | DiLO cards | 9 |
| (16) | Turkey | March 11, 2019 – March 10, 2020 | March 11, 2020 – March 11, 2021 | Breast | Tokat Gaziosmanpasa University (TOGU) Faculty of Medicine General Surgery Clinic | 9.5 |
| (17) | USA | March 1, 2019 – May 31, 2019 | March 1, 2020 – May 31, 2020 | Solid cancer | Montefiore Medical Center | 8.5 |
|  | USA | March 1, 2019 – May 31, 2019 | March 1, 2020 – May 31, 2020 | Hematologic cancer | Montefiore Medical Center | 8.5 |
| (18) | Hong Kong | January 1, 2019 – December 31, 2019 | January 1, 2020 – December 31, 2020 | Miscellaneous | Hong Kong Hospital Authority Clinical Data Analysis and Reporting System (nationwide database) | 9 |
|  | Hong Kong | January 1, 2019 – December 31, 2019 | January 1, 2020 – December 31, 2020 | Colorectal | Hong Kong Hospital Authority Clinical Data Analysis and Reporting System (nationwide database) | 9 |
|  | Hong Kong | January 1, 2019 – December 31, 2019 | January 1, 2020 – December 31, 2020 | Lung | Hong Kong Hospital Authority Clinical Data Analysis and Reporting System (nationwide database) | 9 |
|  | Hong Kong | January 1, 2019 – December 31, 2019 | January 1, 2020 – December 31, 2020 | Breast | Hong Kong Hospital Authority Clinical Data Analysis and Reporting System (nationwide database) | 9 |
|  | Hong Kong | January 1, 2019 – December 31, 2019 | January 1, 2020 – December 31, 2020 | Prostate | Hong Kong Hospital Authority Clinical Data Analysis and Reporting System (nationwide database) | 9 |
| (19) | USA | January 1, 2018 – March 10, 2020 | March 11, 2020 – July 28, 2020 | Breast | Indipendence Bòue Cross for women data | 9.5 |
| (20) | USA | January 1, 2019 – January 31, 2019 | January 1, 2020 – January 31, 2020 | Miscellaneous | COVID and Cancer Research Network (CCRN) | 9 |
|  | USA | February 1, 2019 – February 28, 2019 | February 1, 2020 – February 28, 2020 | Miscellaneous | COVID and Cancer Research Network (CCRN) | 9 |
|  | USA | March 1, 2019 – March 31, 2019 | March 1, 2020 – March 31, 2020 | Miscellaneous | COVID and Cancer Research Network (CCRN) | 9 |
|  | USA | April 1, 2019 – April 30, 2019 | April 1, 2020 – April 30, 2020 | Miscellaneous* | COVID and Cancer Research Network (CCRN) | 9 |
|  | UK | January 1, 2019 – January 31, 2019 | January 1, 2020 – January 31, 2020 | Miscellaneous* | University Hospitals Plymouth NHS Trust | 9 |
|  | UK | February 1, 2019 – February 28, 2019 | February 1, 2020 – February 28, 2020 | Miscellaneous* | University Hospitals Plymouth NHS Trust | 9 |
|  | UK | March 1, 2019 – March 31, 2019 | March 1, 2020 – March 31, 2020 | Miscellaneous | University Hospitals Plymouth NHS Trust | 9 |
|  | UK | April 1, 2019 – April 30, 2019 | April 1, 2020 – April 30, 2020 | Miscellaneous | University Hospitals Plymouth NHS Trust | 9 |
| (21) | UK | June 1, 2019 – November 30, 2019 | June 1, 2020 – November 30, 2020 | Head and neck | Electronic Hospital Records | 9 |
| (22) | USA | March 4, 2019 – May 26, 2019 | March 4, 2020 – May 26, 2020 | Cervix | California Department of Public Health | 8.5 |
| (23) | Japan | January 1, 2017 – February 29, 2020 | March 1, 2020 – December 31, 2020 | Gastrointestinal | Yokohama City University Hospital and the National Hospital Organization Yokohama Medical Center | 9 |
|  | Japan | January 1, 2017 – February 29, 2020 | March 1, 2020 – December 31, 2020 | Colorectal | Yokohama City University Hospital and the National Hospital Organization Yokohama Medical Center | 9 |
|  | Japan | January 1, 2017 – February 29, 2020 | March 1, 2020 – December 31, 2020 | Stomach | Yokohama City University Hospital and the National Hospital Organization Yokohama Medical Center | 9 |
|  | Japan | January 1, 2017 – February 29, 2020 | March 1, 2020 – December 31, 2020 | Pancreas | Yokohama City University Hospital and the National Hospital Organization Yokohama Medical Center | 9 |
|  | Japan | January 1, 2017 – February 29, 2020 | March 1, 2020 – December 31, 2020 | Esophageal | Yokohama City University Hospital and the National Hospital Organization Yokohama Medical Center | 9 |
|  | Japan | January 1, 2017 – February 29, 2020 | March 1, 2020 – December 31, 2020 | Liver | Yokohama City University Hospital and the National Hospital Organization Yokohama Medical Center | 9 |
|  | Japan | January 1, 2017 – February 29, 2020 | March 1, 2020 – December 31, 2020 | Colon | Yokohama City University Hospital and the National Hospital Organization Yokohama Medical Center | 9 |
| (24) | Italy | March 9, 2019 – May 8, 2019 | March 9, 2020 – May 8, 2020 | Lung | Pathological reporting software of the Pathology Units of “Città della Salute e della Scienza di Torino” University Hospital of Turin. | 9 |
|  | Italy | March 9, 2019 – May 8, 2019 | March 9, 2020 – May 8, 2020 | Prostate | Pathological reporting software of the Pathology Units of “Città della Salute e della Scienza di Torino” University Hospital of Turin. | 9 |
|  | Italy | March 9, 2019 – May 8, 2019 | March 9, 2020 – May 8, 2020 | Colorectal | Pathological reporting software of the Pathology Units of “Città della Salute e della Scienza di Torino” University Hospital of Turin. | 9 |
|  | Italy | March 9, 2019 – May 8, 2019 | March 9, 2020 – May 8, 2020 | Breast | Pathological reporting software of the Pathology Units of “Città della Salute e della Scienza di Torino” University Hospital of Turin. | 9 |
| (25) | Saudi Arabia | December 1, 2019 – February 29, 2020 | March 1, 2020 – May 31, 2020 | Solid cancer* | Pediatric oncology and hematopoietics tem cell transplant (HSCT) service in Riyadh | 7 |
| (26) | Slovenia | November 1, 2019 – February 29, 2020 | April 1, 2020 – April 30, 2020 | Miscellaneous* | Slovenian Cancer Registry | 9 |
| (27) | UK | March 1, 2019 – June 30, 2019 | July 1, 2020 – October 31, 2020 | Melanoma | UK Ocular Oncology Services | 7 |
| (28) | USA | March 15, 2019 – July 15, 2019 | March 15, 2020 – July 15, 2020 | Esophago-gastric | Multiple healthcare organizations (HCOs) in the United States | 7 |
|  | USA | March 15, 2019 – July 15, 2019 | March 15, 2020 – July 15, 2020 | Pancreas | Multiple healthcare organizations (HCOs) in the United States | 7 |
|  | USA | March 15, 2019 – July 15, 2019 | March 15, 2020 – July 15, 2020 | Liver | Multiple healthcare organizations (HCOs) in the United States | 7 |
|  | USA | March 15, 2019 – July 15, 2019 | March 15, 2020 – July 15, 2020 | Colorectal | Multiple healthcare organizations (HCOs) in the United States | 7 |
|  | USA | July 16, 2019 – November 15, 2019 | July 16, 2020 – November 15, 2020 | Esophago-gastric | Multiple healthcare organizations (HCOs) in the United States | 7 |
|  | USA | July 16, 2019 – November 15, 2019 | July 16, 2020 – November 15, 2020 | Pancreas | Multiple healthcare organizations (HCOs) in the United States | 7 |
|  | USA | July 16, 2019 – November 15, 2019 | July 16, 2020 – November 15, 2020 | Liver | Multiple healthcare organizations (HCOs) in the United States | 7 |
|  | USA | July 16, 2019 – November 15, 2019 | July 16, 2020 – November 15, 2020 | Colorectal | Multiple healthcare organizations (HCOs) in the United States | 7 |
| (29) | UK | January 6, 2020 – March 15, 2020 | March 23, 2020 – May 31, 2020 | Colorectal | UK's National Endoscopy Database (NED) | 7.5 |
| (30) | Netherlands | January 8, 2019 – September 2, 2018 | January 6, 2020 – August 30, 2020 | Breast | Netherlands Cancer Registry | 8 |
|  | Netherlands | July 1, 2019 – September 1, 2019 | January 6, 2020 – August 30, 2020 | Breast | Netherlands Cancer Registry | 8 |
| (31) | Spain | April 1, 2019 – August 31, 2019 | April 1, 2020 – August 31, 2020 | Melanoma | Costa del Sol Hospital | 7 |
| (32) | China | October 1, 2019 – January 21, 2020 | January 27, 2020 – March 31, 2020 | Colorectal | Clinical Data Analysis and Reporting System of the Hong Kong Hospital Authority | 8 |
|  | China | October 1, 2019 – January 21, 2020 | January 27, 2020 – March 31, 2020 | Stomach | Clinical Data Analysis and Reporting System of the Hong Kong Hospital Authority | 8 |
| (33) | Northern Ireland | March 1, 2019 – September 12, 2019 | March 1, 2020 – September 12, 2020 | Miscellaneous* | Northern Ireland Cancer Registry (NICR) | 7 |
|  | Northern Ireland | March 1, 2019 – September 12, 2019 | March 1, 2020 – September 12, 2020 | Colorectal* | Northern Ireland Cancer Registry (NICR) | 7 |
|  | Northern Ireland | March 1, 2019 – September 12, 2019 | March 1, 2020 – September 12, 2020 | Lung* | Northern Ireland Cancer Registry (NICR) | 7 |
|  | Northern Ireland | March 1, 2019 – September 12, 2019 | March 1, 2020 – September 12, 2020 | Breast* | Northern Ireland Cancer Registry (NICR) | 7 |
|  | Northern Ireland | March 1, 2019 – September 12, 2019 | March 1, 2020 – September 12, 2020 | Prostate* | Northern Ireland Cancer Registry (NICR) | 7 |
|  | Northern Ireland | March 1, 2019 – September 12, 2019 | March 1, 2020 – September 12, 2020 | Melanoma* | Northern Ireland Cancer Registry (NICR) | 7 |
| (34) | Italy | January 1, 2019 – December 31, 2019 | March 11, 2020 – April 6, 2020 | Breast | Breast Unit of OspedaleSanta Chiara, Trento | 9 |
| (35) | France | January 20, 2020 – March 15, 2020 | March 16, 2020 – May 10, 2020 | Miscellaneous | Oscar Lambret Cancer center, Northern France | 9 |
|  | France | January 20, 2020 – March 15, 2020 | May 11, 2020 – July 06, 2020 | Miscellaneous | Oscar Lambret Cancer center, Northern France | 9 |
| (36) | Belgium | March 17, 2019 – August 28, 2019 | March 17, 2020 – August 28, 2020 | Breast | Antwerp University Hospital Cancer Center (MOCA) | 8 |
|  | Belgium | March 17, 2019 – August 28, 2019 | March 17, 2020 – August 28, 2020 | Colorectal | Antwerp University Hospital Cancer Center (MOCA) | 8 |
|  | Belgium | March 17, 2019 – August 28, 2019 | March 17, 2020 – August 28, 2020 | Lung | Antwerp University Hospital Cancer Center (MOCA) | 8 |
|  | Belgium | March 17, 2019 – August 28, 2019 | March 17, 2020 – August 28, 2020 | Prostate | Antwerp University Hospital Cancer Center (MOCA) | 8 |
| (37) | Singapore | January 1, 2019 – October 31, 2019 | January 1, 2020 – October 31, 2020 | Colorectal | National University of Singapore cohort | 8 |
| (38) | France | January 1, 2019 – September 30, 2019 | January 1, 2020 – September 30, 2020 | Miscellaneous | Assistance Publique Hôpitaux de Paris Teaching Hospital | 10 |
|  | France | January 1, 2019 – January 31, 2019 | January 1, 2020 - January 31, 2020 | Miscellaneous | Assistance Publique Hôpitaux de Paris Teaching Hospital | 10 |
|  | France | February 1, 2019 - 28, 2019 | February 1, 2020 - February 29, 2020 | Miscellaneous | Assistance Publique Hôpitaux de Paris Teaching Hospital | 10 |
|  | France | March 1, 2019 – March 31, 2019 | March 1, 2020 – March 31, 2020 | Miscellaneous | Assistance Publique Hôpitaux de Paris Teaching Hospital | 10 |
|  | France | April 1, 2019 – April 30, 2019 | April 1, 2020 – April 30, 2020 | Miscellaneous | Assistance Publique Hôpitaux de Paris Teaching Hospital | 10 |
|  | France | May 1, 2019 – May 31, 2019 | May 1, 2020 – May 31, 2020 | Miscellaneous | Assistance Publique Hôpitaux de Paris Teaching Hospital | 10 |
|  | France | June 1, 2019 – June 30, 2019 | June 1, 2020 – June 30, 2020 | Miscellaneous | Assistance Publique Hôpitaux de Paris Teaching Hospital | 10 |
|  | France | July 1, 2019 – July 31, 2019 | July 1, 2020 – July 31, 2020 | Miscellaneous | Assistance Publique Hôpitaux de Paris Teaching Hospital | 10 |
|  | France | August 1, 2019 – August 31, 2019 | August 1, 2020 – August 31, 2020 | Miscellaneous | Assistance Publique Hôpitaux de Paris Teaching Hospital | 10 |
|  | France | September 1, 2019 – September 30, 2019 | September 1, 2020 – September 30, 2020 | Miscellaneous | Assistance Publique Hôpitaux de Paris Teaching Hospital | 10 |
|  | France | March 1, 2019 – May 31, 2019 | March 1, 2020 – May 31, 2020 | Colorectal | Assistance Publique Hôpitaux de Paris Teaching Hospital | 10 |
|  | France | March 1, 2019 – May 31, 2019 | March 1, 2020 – May 31, 2020 | Cervix | Assistance Publique Hôpitaux de Paris Teaching Hospital | 10 |
|  | France | March 1, 2019 – May 31, 2019 | March 1, 2020 – May 31, 2020 | Breast | Assistance Publique Hôpitaux de Paris Teaching Hospital | 10 |
|  | France | March 01, 2019 – May 31, 2019 | March 01, 2020 – May 31, 2020 | Lung | Assistance Publique Hôpitaux de Paris Teaching Hospital | 10 |
|  | France | March 1, 2019 – May 31, 2019 | March 1, 2020 – May 31, 2020 | Prostate | Assistance Publique Hôpitaux de Paris Teaching Hospital | 10 |
|  | France | March 1, 2019 – May 31, 2019 | March 1, 2020 – May 31, 2020 | Melanoma | Assistance Publique Hôpitaux de Paris Teaching Hospital | 10 |
|  | France | June 1, 2019 – September 30, 2019 | June 1, 2020 – September 30, 2020 | Colorectal | Assistance Publique Hôpitaux de Paris Teaching Hospital | 10 |
|  | France | June 1, 2019 – September 30, 2019 | June 1, 2020 – September 30, 2020 | Cervix | Assistance Publique Hôpitaux de Paris Teaching Hospital | 10 |
|  | France | June 1, 2019 – September 30, 2019 | June 1, 2020 – September 30, 2020 | Breast | Assistance Publique Hôpitaux de Paris Teaching Hospital | 10 |
|  | France | June 1, 2019 – September 30, 2019 | June 1, 2020 – September 30, 2020 | Lung | Assistance Publique Hôpitaux de Paris Teaching Hospital | 10 |
|  | France | June 1, 2019 – September 30, 2019 | June 1, 2020 – September 30, 2020 | Prostate | Assistance Publique Hôpitaux de Paris Teaching Hospital | 10 |
|  | France | June 1, 2019 – September 30, 2019 | June 1, 2020 – September 30, 2020 | Melanoma | Assistance Publique Hôpitaux de Paris Teaching Hospital | 10 |
| (39) | India | February 10, 2020 – March 23, 2020 | March 24, 2020 – May 05, 2020 | Miscellaneous | North India University Hospital | 7.5 |
| (40) | USA | January 1, 2018 – February 29, 2020 | March 1, 2020 – May 31, 2020 | Prostate | Quest diagnostic database | 10 |
|  | USA | January 1, 2018 – February 29, 2020 | June 1, 2020 – December 31, 2020 | Prostate | Quest diagnostic database | 10 |
| (41) | USA | January 1, 2019 – March 2, 2020 | March 3, 2020 – May 21, 2020 | Breast | 7 breast imaging facilities in the University of North Carolina (UNC) Health system | 10 |
|  | USA | January 1, 2019 – March 2, 2020 | May 22, 2020 – September 30, 2020 | Breast | 7 breast imaging facilities in the University of North Carolina (UNC) Health system | 10 |
| (42) | Sweden | March 18, 2019 – June 2, 2019 | March 18, 2020 – June 2, 2020 | Prostate | National Prostate Cancer Register (NPCR) of Sweden | 9.5 |
| (43) | USA | December 1, 2019 – March 2, 2020 | March 2, 2020 – 2 June 2020 | Miscellaneous | Massachusetts General Brigham (MGB) Healthcare System | 8.5 |
|  | USA | December 1, 2019 – March 2, 2020 | September 4, 2020 – December 5, 2020 | Colorectal* | Massachusetts General Brigham (MGB) Healthcare System | 8.5 |
| (44) | UK | March 23, 2019 – June 23, 2019 | March 23, 2020 – June 23, 2020 | Skin cancer | The Northern Cancer Network | 8 |
| (45) | USA | December 17, 2019 – March 16, 2020 | March 17, 2020 – June 17, 2020 | Genito-urinary | Electronic medical record, Pittsburgh | 9.5 |
|  | USA | December 17, 2019 – March 16, 2020 | March 17, 2020 – June 17, 2020 | Prostate | Electronic medical record, Pittsburgh | 9.5 |
|  | USA | December 17, 2019 – March 16, 2020 | March 17, 2020 – June 17, 2020 | Bladder | Electronic medical record, Pittsburgh | 9.5 |
|  | USA | December 17, 2019 – March 16, 2020 | March 17, 2020 – June 17, 2020 | Kidney | Electronic medical record, Pittsburgh | 9.5 |
| (46) | Australia | January 1, 2019 – March 31, 2019 | January 1, 2020 – March 31, 2020 | Skin cancer* | MedicineInsight and the national Medicare Benefits Schedule (MBS) service | 7.5 |
|  | Australia | January 1, 2019 – March 31, 2019 | January 1, 2020 – March 31, 2020 | Melanoma* | MedicineInsight and the national Medicare Benefits Schedule (MBS) service | 7.5 |
| (47) | USA | May 16, 2019 – June 20, 2019 | May 14, 2020 – June 18, 2020 | Head & neck* | The Head and Neck multidisciplinary tumor conference (MTC) at the University of Texas M. D. Anderson Cancer Center (UTMDACC) | 9 |
| (48) | Northern Ireland | March 1, 2019 – September 30, 2019 | March 1, 2020 – September 30, 2020 | Solid cancer* | The Northern Ireland Cancer Registry (NICR) | 9.5 |
| (49) | Netherlands | January 1, 2019 – November 30, 2019 | January 1, 2020 – November 30, 2020 | Colorectal* | Dutch CRC screening registry | 9 |
| (50) | USA | March 1, 2019 – March 31, 2019 | March 1, 2020 – March 31, 2020 | Melanoma | Outpatient-chart reviews of US dermatology practices | 7.5 |
|  | USA | March 1, 2019 – March 31, 2019 | March 1, 2020 – March 31, 2020 | Squamous Cell Carcinoma | Outpatient-chart reviews of US dermatology practices | 7.5 |
|  | USA | March 1, 2019 – March 31, 2019 | March 1, 2020 – March 31, 2020 | Basal Cell Carcinoma | Outpatient-chart reviews of US dermatology practices | 7.5 |
|  | USA | April 1, 2019 – April 30, 2019 | April 1, 2020 – April 30, 2020 | Melanoma | Outpatient-chart reviews of US dermatology practices | 7.5 |
|  | USA | April 1, 2019 – April 30, 2019 | April 1, 2020 – April 30, 2020 | Squamous Cell Carcinoma | Outpatient-chart reviews of US dermatology practices | 7.5 |
|  | USA | April 1, 2019 – April 30, 2019 | April 1, 2020 – April 30, 2020 | Basal Cell Carcinoma | Outpatient-chart reviews of US dermatology practices | 7.5 |
|  | USA | May 1, 2019 – May 31, 2019 | May 1, 2020 – May 31, 2020 | Melanoma | Outpatient-chart reviews of US dermatology practices | 7.5 |
|  | USA | May 1, 2019 – May 31, 2019 | May 1, 2020 – May 31, 2020 | Squamous Cell Carcinoma | Outpatient-chart reviews of US dermatology practices | 7.5 |
|  | USA | May 1, 2019 – May 31, 2019 | May 1, 2020 – May 31, 2020 | Basal Cell Carcinoma | Outpatient-chart reviews of US dermatology practices | 7.5 |
|  | USA | June 1, 2019 – June 30, 2019 | June 1, 2020 – June 30, 2020 | Melanoma | Outpatient-chart reviews of US dermatology practices | 7.5 |
|  | USA | June 1, 2019 – June 30, 2019 | June 1, 2020 – June 30, 2020 | Squamous Cell Carcinoma | Outpatient-chart reviews of US dermatology practices | 7.5 |
|  | USA | June 1, 2019 – June 30, 2019 | June 1, 2020 – June 30, 2020 | Basal Cell Carcinoma | Outpatient-chart reviews of US dermatology practices | 7.5 |
|  | USA | July 1, 2019 – July 31, 2019 | July 1, 2020 – July 31, 2020 | Melanoma | Outpatient-chart reviews of US dermatology practices | 7.5 |
|  | USA | July 1, 2019 – July 31, 2019 | July 1, 2020 – July 31, 2020 | Squamous Cell Carcinoma | Outpatient-chart reviews of US dermatology practices | 7.5 |
|  | USA | July 1, 2019 – July 31, 2019 | July 1, 2020 – July 31, 2020 | Basal Cell Carcinoma | Outpatient-chart reviews of US dermatology practices | 7.5 |
|  | USA | August 1, 2019 – August 31, 2019 | August 1, 2020 – August 31, 2020 | Melanoma | Outpatient-chart reviews of US dermatology practices | 7.5 |
|  | USA | August 1, 2019 – August 31, 2019 | August 1, 2020 – August 31, 2020 | Squamous Cell Carcinoma | Outpatient-chart reviews of US dermatology practices | 7.5 |
|  | USA | August 1, 2019 – August 31, 2019 | August 1, 2020 – August 31, 2020 | Basal Cell Carcinoma | Outpatient-chart reviews of US dermatology practices | 7.5 |
| (51) | Canada | January 07, 2019 – April 21, 2019 | January 06, 2020 – April 19, 2020 | Melanoma | Universal health care claims dataset from Ontario | 7 |

**Supplementary Table 3 Characteristics of studies selected for cancer diagnostic tests**

| **Reference** | **Country** | **Contrast period** | **Period of study** | **Site of cancer** | **Setting and source of data** | **Quality score** |
| --- | --- | --- | --- | --- | --- | --- |
| (4) | USA | March 18, 2019 – April 24, 2019 | March 18, 2020 – April 24, 2020 | Prostate | EHR data from Vanderbilt University Medical Center’s (VUMC) Research and Synthetic Derivatives | 9.5 |
| (51) | Canada | January 07, 2019 – April 21, 2019 | January 06, 2020 – April 19, 2020 | Melanoma | Universal health care claims dataset from Ontario | 7 |
|  | Canada | January 07, 2019 – April 21, 2019 | January 06, 2020 – April 19, 2020 | Skin cancer | Universal health care claims dataset from Ontario | 7 |
| (52) | Italy | January 01, 2019 – March 13, 2020 | March 14, 2020 – May 15, 2020 | Thyroid | Federico II University Hospital, Naples | 9.5 |
|  | Italy | January 01, 2019 – March 13, 2020 | May 16, 2020 – July 07, 2020 | Thyroid | Federico II University Hospital, Naples | 9.5 |
| (53) | Belgium | March 1, 2019 – March 31, 2019 | March 1, 2020 – March 31, 2020 | Miscellaneous* | Department of Pathology of the Cliniques universitaires Saint-Luc, Brussels | 9 |
|  | Belgium | April 1, 2019 – April 30, 2019 | April 1, 2020 – April 30, 2020 | Miscellaneous* | Department of Pathology of the Cliniques universitaires Saint-Luc, Brussels | 9 |
|  | Belgium | March 1, 2019 – March 31, 2019 | March 1, 2020 – March 31, 2020 | Colorectal | Department of Pathology of the Cliniques universitaires Saint-Luc, Brussels | 9 |
|  | Belgium | April 1, 2019 – April 30, 2019 | April 1, 2020 – April 30, 2020 | Colorectal | Department of Pathology of the Cliniques universitaires Saint-Luc, Brussels | 9 |
|  | Belgium | March 1, 2019 – March 31, 2019 | March 1, 2020 – March 31, 2020 | Stomach | Department of Pathology of the Cliniques universitaires Saint-Luc, Brussels | 9 |
|  | Belgium | April 1, 2019 – April 30, 2019 | April 1, 2020 – April 30, 2020 | Stomach | Department of Pathology of the Cliniques universitaires Saint-Luc, Brussels | 9 |
|  | Belgium | March 1, 2019 – March 31, 2019 | March 1, 2020 – March 31, 2020 | Cervix | Department of Pathology of the Cliniques universitaires Saint-Luc, Brussels | 9 |
|  | Belgium | April 1, 2019 – April 30, 2019 | April 1, 2020 – April 30, 2020 | Cervix | Department of Pathology of the Cliniques universitaires Saint-Luc, Brussels | 9 |
|  | Belgium | March 1, 2019 – March 31, 2019 | March 1, 2020 – March 31, 2020 | Skin cancer | Department of Pathology of the Cliniques universitaires Saint-Luc, Brussels | 9 |
|  | Belgium | April 1, 2019 – April 30, 2019 | April 1, 2020 – April 30, 2020 | Skin cancer | Department of Pathology of the Cliniques universitaires Saint-Luc, Brussels | 9 |
|  | Belgium | March 1, 2019 – March 31, 2019 | March 1, 2020 – March 31, 2020 | Prostate | Department of Pathology of the Cliniques universitaires Saint-Luc, Brussels | 9 |
|  | Belgium | March 1, 2019 – March 31, 2019 | March 1, 2020 – March 31, 2020 | Breast | Department of Pathology of the Cliniques universitaires Saint-Luc, Brussels | 9 |
|  | Belgium | April 1, 2019 – April 30, 2019 | April 1, 2020 – April 30, 2020 | Breast | Department of Pathology of the Cliniques universitaires Saint-Luc, Brussels | 9 |
|  | Belgium | March 1, 2019 – March 31, 2019 | March 1, 2020 – March 31, 2020 | Lung | Department of Pathology of the Cliniques universitaires Saint-Luc, Brussels | 9 |
|  | Belgium | April 1, 2019 – April 30, 2019 | April 1, 2020 – April 30, 2020 | Lung | Department of Pathology of the Cliniques universitaires Saint-Luc, Brussels | 9 |
|  | Belgium | March 1, 2019 – March 31, 2019 | March 1, 2020 – March 31, 2020 | CNS | Department of Pathology of the Cliniques universitaires Saint-Luc, Brussels | 9 |
|  | Belgium | April 1, 2019 – April 30, 2019 | April 1, 2020 – April 30, 2020 | CNS | Department of Pathology of the Cliniques universitaires Saint-Luc, Brussels | 9 |
|  | Belgium | March 1, 2019 – March 31, 2019 | March 1, 2020 – March 31, 2020 | Breast | Department of Pathology of the Cliniques universitaires Saint-Luc, Brussels | 9 |
|  | Belgium | April 1, 2019 – April 30, 2019 | April 1, 2020 – April 30, 2020 | Breast | Department of Pathology of the Cliniques universitaires Saint-Luc, Brussels | 9 |
| (18) | Hong Kong | January 1, 2019 – December 31, 2019 | January 1, 2020 – December 31, 2020 | Miscellaneous | Hong Kong Hospital Authority Clinical Data Analysis and Reporting System (nationwide database) | 9 |
|  | Hong Kong | January 1, 2019 – December 31, 2019 | January 1, 2020 – December 31, 2020 | Colorectal | Hong Kong Hospital Authority Clinical Data Analysis and Reporting System (nationwide database) | 9 |
|  | Hong Kong | January 1, 2019 – December 31, 2019 | January 1, 2020 – December 31, 2020 | Lung | Hong Kong Hospital Authority Clinical Data Analysis and Reporting System (nationwide database) | 9 |
|  | Hong Kong | January 1, 2019 – December 31, 2019 | January 1, 2020 – December 31, 2020 | Breast | Hong Kong Hospital Authority Clinical Data Analysis and Reporting System (nationwide database) | 9 |
|  | Hong Kong | January 1, 2019 – December 31, 2019 | January 1, 2020 – December 31, 2020 | Prostate | Hong Kong Hospital Authority Clinical Data Analysis and Reporting System (nationwide database) | 9 |
| (13) | Italy | March 1, 2019 – December 31, 2019 | March 1, 2020 – December 31, 2020 | Head and Neck | Department of Oral and Maxillofacial Sciences. Sapienza University of  Rome and Umberto I of Rome | 8 |
| (54) | India | March 1, 2019 – May 31, 2019 | March 1, 2020 – May 31, 2020 | Miscellaneous | National Cancer Grid of India | 10 |
|  | India | March 1, 2019 – May 31, 2019 | March 1, 2020 – May 31, 2020 | Miscellaneous | National Cancer Grid of India | 10 |
| (55) | USA | March 1, 2019 – March 31, 2019 | March 1, 2020 – March 31, 2020 | Breast* | Proprietary provider clearinghouse registry | 9.5 |
|  | USA | March 1, 2019 – March 31, 2019 | March 1, 2020 – March 31, 2020 | Colorectal* | Proprietary provider clearinghouse registry | 9.5 |
|  | USA | March 1, 2019 – March 31, 2019 | March 1, 2020 – March 31, 2020 | Lung* | Proprietary provider clearinghouse registry | 9.5 |
|  | USA | July 1, 2019 – July 31, 2019 | July 1, 2020 – July 31, 2020 | Breast* | Proprietary provider clearinghouse registry | 9.5 |
|  | USA | April 1, 2019 – April 30, 2019 | April 1, 2020 – April 30, 2020 | Breast* | Proprietary provider clearinghouse registry | 9.5 |
|  | USA | April 1, 2019 – April 30, 2019 | April 1, 2020 – April 30, 2020 | Colorectal* | Proprietary provider clearinghouse registry | 9.5 |
|  | USA | April 1, 2019 – April 30, 2019 | April 1, 2020 – April 30, 2020 | Lung* | Proprietary provider clearinghouse registry | 9.5 |
|  | USA | July 1, 2019 – July 31, 2019 | July 1, 2020 – July 31, 2020 | Colorectal* | Proprietary provider clearinghouse registry | 9.5 |
|  | USA | May 1, 2019 – May 31, 2019 | May 1, 2020 – May 31, 2020 | Breast* | Proprietary provider clearinghouse registry | 9.5 |
|  | USA | May 1, 2019 – May 31, 2019 | May 1, 2020 – May 31, 2020 | Colorectal* | Proprietary provider clearinghouse registry | 9.5 |
|  | USA | May 1, 2019 – May 31, 2019 | May 1, 2020 – May 31, 2020 | Lung* | Proprietary provider clearinghouse registry | 9.5 |
|  | USA | July 1, 2019 – July 31, 2019 | July 1, 2020 – July 31, 2020 | Lung* | Proprietary provider clearinghouse registry | 9.5 |
|  | USA | June 1, 2019 – June 30, 2019 | June1, 2020 – June 30, 2020 | Breast* | Proprietary provider clearinghouse registry | 9.5 |
|  | USA | June 1, 2019 – June 30, 2019 | June1, 2020 – June 30, 2020 | Colorectal* | Proprietary provider clearinghouse registry | 9.5 |
|  | USA | June 1, 2019 – June 30, 2019 | June1, 2020 – June 30, 2020 | Lung* | Proprietary provider clearinghouse registry | 9.5 |
| (56) | USA | January 5, 2020 – March 14, 2020 | March 15, 2020 – May 2, 2020 | Miscellaneous* | Urban quaternary academic center (QAC) and three affiliated community medical centers in Massachusetts | 10 |
|  | USA | January 5, 2020 – March 14, 2020 | May 5, 2020 – November 14, 2020 | Miscellaneous* | Urban quaternary academic center (QAC) and three affiliated community medical centers in Massachusetts | 10 |
| (57) | Canada | June 1, 2019 – August 31, 2019 | June 1, 2020 – August 31, 2020 | Cervix* | CytoBase | 9.5 |
|  | Canada | March 1, 2019 – March 31, 2019 | March 1, 2020 – March 31, 2020 | Cervix* | CytoBase | 9.5 |
|  | Canada | April 1, 2019 – April 30, 2019 | April 1, 2020 – April 30, 2020 | Cervix* | CytoBase | 9.5 |
|  | Canada | May 1, 2019 – May 31, 2019 | May 1, 2020 – May 31, 2020 | Cervix* | CytoBase | 9.5 |
| (58) | Uruguay | March 16, 2019 – June 30, 2019 | March 16, 2020 – June 30, 2020 | Miscellaneous* | Public and Private sector | 9.5 |
|  | Chile | March 16, 2019 – April 30, 2019 | March 16, 2020 – April 30, 2020 | Miscellaneous* | National Cancer Institute | 9.5 |
|  | Chile | May 1, 2019 – May 31, 2019 | May 1, 2020 – May 31, 2020 | Miscellaneous* | National Cancer Institute | 9.5 |
|  | Chile | June 01, 2019 – June 30, 2019 | June 01, 2020 – June 30, 2020 | Miscellaneous* | National Cancer Institute | 9.5 |
|  | Mexico | April 01, 2019 – April 30, 2019 | April 01, 20120 – April 30, 2020 | Miscellaneous* | General Hospital | 9.5 |
|  | Mexico | May 1, 2019 – May 31, 2019 | May 1, 2020 – May 31, 2020 | Miscellaneous* | General Hospital | 9.5 |
|  | Mexico | June 01, 2019 – June 30, 2019 | June 01, 2020 – June 30, 2020 | Miscellaneous* | General Hospital | 9.5 |
|  | Mexico | April 01, 2019 – April 30, 2019 | April 01, 20120 – April 30, 2020 | Miscellaneous* | National Cancer Institute | 9.5 |
|  | Mexico | May 1, 2019 – May 31, 2019 | May 1, 2020 – May 31, 2020 | Miscellaneous* | National Cancer Institute | 9.5 |
|  | Mexico | June 01, 2019 – June 30, 2019 | June 01, 2020 – June 30, 2020 | Miscellaneous* | National Cancer Institute | 9.5 |
|  | Perù | March 16, 2019 – June 30, 2019 | March 16, 2020 – June 30, 2020 | Miscellaneous* | Private Health Provider | 9.5 |
|  | Brazil | March 16, 2019 – April 30, 2019 | March 16, 2020 – April 30, 2020 | Miscellaneous* | Public Health Provider | 9.5 |
|  | Brazil | May 1, 2019 – May 31, 2019 | May 1, 2020 – May 31, 2020 | Miscellaneous* | Public Health Provider | 9.5 |
|  | Brazil | June 01, 2019 – June 30, 2019 | June 01, 2020 – June 30, 2020 | Miscellaneous* | Public Health Provider | 9.5 |
| (59) | Brazil | March 1, 2019 – May 31, 2019 | March 1, 2020 – May 31, 2020 | Miscellaneous* | Brasil’s National Health System | 9 |
|  | Brazil | March 1, 2019 – May 31, 2019 | March 1, 2020 – May 31, 2020 | Colorectal* | Brasil’s National Health System | 9 |
|  | Brazil | March 1, 2019 – May 31, 2019 | March 1, 2020 – May 31, 2020 | Miscellaneous* | Brasil’s National Health System | 9 |
|  | Brazil | March 1, 2019 – May 31, 2019 | March 1, 2020 – May 31, 2020 | Breast* | Brasil’s National Health System | 9 |
| (60) | France | March 1, 2019 – May 31, 2019 | March 1, 2020 – May 31, 2020 | Miscellaneous* | Grand East region, University Hospital and Godinot Cancer Institute in Reims, Colmae General Hospital | 9.5 |
| (61) | Germany | January 1, 2019 – December 31, 2019 | April 1, 2020 – April 30, 2020 | Miscellaneous | North Rhine-Westphalia State Cancer Registry | 8 |

**Supplementary Table 4 Selected studies on differences of cancer’s characteristics at diagnosis in the pre-COVID-19 (reference period) and COVID-19 pandemic (study period).**

| **Reference** | **Country** | **Reference period** | **Study period** | **Site of cancer** | **Outcome** | **Percentage on the total diagnosis in the contrast period** | **Percentage on the total diagnosis in the period on exam** |
| --- | --- | --- | --- | --- | --- | --- | --- |
| (8) | Italy | March 11, 2019 – May 19, 2019 | March 9, 2020 – May 17, 2020 | Prostate | Grade Group 4-5 | 13.8%* | 50.0%* |
| (16) | Turkey | March 11, 2019 – March 10, 2020 | March 11, 2020 – March 11, 2021 | Breast | T1 | 25.7% | 5.1% |
|  |  |  |  |  | T2 | 37.5% | 47.4% |
|  |  |  |  |  | T3 | 31.4% | 34.6% |
|  |  |  |  |  | T4 | 5.7% | 12.8% |
|  |  |  |  |  | Axillary involvement | 35.7% | 56.4% |
|  |  |  |  |  | No axillary involvement | 64.3% | 43.6% |
|  |  |  |  |  | Metastasis | 2.9% | 2.6% |
|  |  |  |  |  | No metastasis | 97.1% | 97.4% |
| (1) | Netherlands | January 7, 2019 – April 28, 2019 | January 6, 2020 – April 26, 2020 | Breast | DCIS | 11.9%* | 12.4%* |
|  |  |  |  |  | Stage I | 41.0%* | 39.6%* |
|  |  |  |  |  | Stage II | 33.2%* | 34.2%* |
|  |  |  |  |  | Stage III | 9.1%* | 8.2%* |
|  |  |  |  |  | Stage IV | 4.8%* | 5.6%* |
| (21) | UK | June 1, 2019 – November 30, 2019 | June 1, 2020 – November 30, 2020 | Head and Neck | T3-T4 (advanced primary disease) | 90.9% | 93.1% |
| (23) | Japan | January 1, 2017 – February 29, 2020 | March 1, 2020 – December 31, 2020 | Colorectal cancer | 0 | 25,4%* | 19.7%* |
|  |  |  |  | Colorectal cancer | I | 24.4%* | 18.6%* |
|  |  |  |  | Colorectal cancer | II | 17.8%* | 13.3%* |
|  |  |  |  | Colorectal cancer | III | 17.3%* | 33.6%* |
|  |  |  |  | Colorectal cancer | IV | 15.0%* | 14.7%* |
|  |  |  |  | Gastric cancer | I | 70.3%* | 62.1%* |
|  |  |  |  | Gastric cancer | II | 8.8%* | 9.8%* |
|  |  |  |  | Gastric cancer | III | 6.4%* | 6.3%* |
|  |  |  |  | Gastric cancer | IV | 14.3%* | 21.9%* |
|  |  |  |  | Pancreatic cancer | 0 | 3.6%* | 5.7%* |
|  |  |  |  | Pancreatic cancer | I | 23.9%* | 17.7%* |
|  |  |  |  | Pancreatic cancer | II | 17.5%* | 17.0%* |
|  |  |  |  | Pancreatic cancer | III | 14.8%* | 16.3%* |
|  |  |  |  | Pancreatic cancer | IV | 40.2%* | 43.3%* |
|  |  |  |  | Esophageal cancer | 0 | 14.6%* | 10.3%* |
|  |  |  |  | Esophageal cancer | I | 35.8%* | 35.6%* |
|  |  |  |  | Esophageal cancer | II | 11.0%* | 9.2%* |
|  |  |  |  | Esophageal cancer | III | 14.9%* | 17.2%* |
|  |  |  |  | Esophageal cancer | IV | 23.6%* | 27.6%* |
|  |  |  |  | Hepatocellular carcinoma | I | 54.7%* | 44.0%* |
|  |  |  |  | Hepatocellular carcinoma | II | 20.7%* | 22.7%* |
|  |  |  |  | Hepatocellular carcinoma | III | 15.1%* | 21.3%* |
|  |  |  |  | Hepatocellular carcinoma | IV | 9.5%* | 12.0%* |
|  |  |  |  | Biliary tract cancer | 0 | 4.9%* | 4.8%* |
|  |  |  |  | Biliary tract cancer | I | 18.3%* | 17.7%* |
|  |  |  |  | Biliary tract cancer | II | 26.1%* | 37.1%* |
|  |  |  |  | Biliary tract cancer | III | 22.0%* | 19.4%* |
|  |  |  |  | Biliary tract cancer | IV | 28.7%* | 21.0%* |
| (24) | Italy | March 9, 2019 – May 8, 2019 | March 9, 2020 – May 8, 2020 | Lung | T1 | 34.6%* | 28.1%* |
|  |  |  |  | Lung | T2 | 30.8%* | 37.5%* |
|  |  |  |  | Lung | T3 | 23.1%* | 15.6%* |
|  |  |  |  | Lung | T4 | 11.5%* | 18.8%* |
|  |  |  |  | Pancreas | T1 | 9.1%* | 31.3%* |
|  |  |  |  | Pancreas | T2 | 27.3%* | 43.8%* |
|  |  |  |  | Pancreas | T3 | 63.6%* | 25.0%* |
|  |  |  |  | Pancreas | T4 | 0%* | 0%* |
|  |  |  |  | Prostate  Prostate | T2 | 53.1%* | 48.1%* |
|  |  |  |  | Prostate | T3 | 46.9%* | 51.9%* |
|  |  |  |  | Prostate | T4 | 0%* | 0%* |
|  |  |  |  | Uterus | Tis | 0%* | 4.2%* |
|  |  |  |  | Uterus | T1 | 68.4%* | 83.3%* |
|  |  |  |  | Uterus | T2 | 15.7%* | 4.2%* |
|  |  |  |  | Uterus | T3 | 15.7%* | 8.3%* |
|  |  |  |  | Uterus | T4 | 0%* | 0%* |
|  |  |  |  | Ovary | T1 | 16.7%* | 28.6%* |
|  |  |  |  | Ovary | T2 | 33.3%* | 14.3%* |
|  |  |  |  | Ovary | T3 | 50.0%* | 57.1%* |
|  |  |  |  | Ovary | T4 | 0%* | 0%* |
|  |  |  |  | Colon | Tis | 0%* | 0%* |
|  |  |  |  | Colon | T1 | 5.6%* | 4.2%* |
|  |  |  |  | Colon | T2 | 18.5%* | 12.5%* |
|  |  |  |  | Colon | T3 | 61.1%* | 56.3%* |
|  |  |  |  | Colon | T4 | 14.8%* | 27.1%* |
|  |  |  |  | Thyroid | T1 | 75.0%* | 66.7%* |
|  |  |  |  | Thyroid | T2 | 12.5%* | 22.2%* |
|  |  |  |  | Thyroid | T3 | 12.5%* | 11.1%* |
|  |  |  |  | Thyroid | T4 | 0%* | 0%* |
|  |  |  |  | CNS | Grade I (gliomas) | 23.8%* | 13.0%* |
|  |  |  |  | CNS | Grade II (gliomas) | 23.8%* | 21.7%* |
|  |  |  |  | CNS | Grade III (gliomas) | 14.3%* | 13.0%* |
|  |  |  |  | CNS | Grade IV (gliomas) | 38.1%* | 52.2%* |
|  |  |  |  | CNS | Grade I (meningiomas) | 86.4%* | 77.3%* |
|  |  |  |  | CNS | Grade II (meningiomas) | 9.1%* | 13.6%* |
|  |  |  |  | CNS | Grade III (meningiomas) | 4.5%* | 9.1%* |
|  |  |  |  | Breast | Tis | 14.5%* | 14.7%* |
|  |  |  |  | Breast | T1 | 58.9%* | 61.8%* |
|  |  |  |  | Breast | T2 | 23.4%* | 21.6%* |
|  |  |  |  | Breast | T3 | 2.4%* | 2.0%* |
|  |  |  |  | Breast | T4 | 0.8%* | 0%* |
| (27) | UK | March 1, 2019 – June 30, 2019 | July 1, 2020 – October 31, 2020 | Uveal melanoma | Stage III-IV | 13.4% | 28.2% |
| (31) | Spain | April 1, 2019 – August 31, 2019 | April 1, 2020 – August 31, 2020 | Melanoma | in situ | 68.8% | 27.8% |
|  |  |  |  |  | thickness >2 mm | 8.3% | 38.9% |
|  |  |  |  |  | ulcerated | 7.1% | 21.4% |
|  |  |  |  |  | presence of mitosis | 42.9% | 75.0% |
| (47) | USA | May 16, 2019 – June 20, 2019 | May 14, 2020 – June 18, 2020 | Head and Neck | I | 39.0% | 33.8% |
|  |  |  |  |  | II | 21.9% | 27.0% |
|  |  |  |  |  | III | 20.0% | 17.6% |
|  |  |  |  |  | IV | 19.0% | 21.6% |
| (52) | Italy | January 01, 2019 – March 13, 2020 | March 14, 2020 – May 15, 2020 | Thyroid | Benign | 72.0% | 55.6% |
|  |  |  |  |  | Malignant or suspicious for malignancy | 6.0% | 11.1% |
| *calculated value using data present in the article | | | | | | | |

**Supplementary Figure 1** Representation of geographic areas of the studies included in the analysis for cancer diagnostic tests


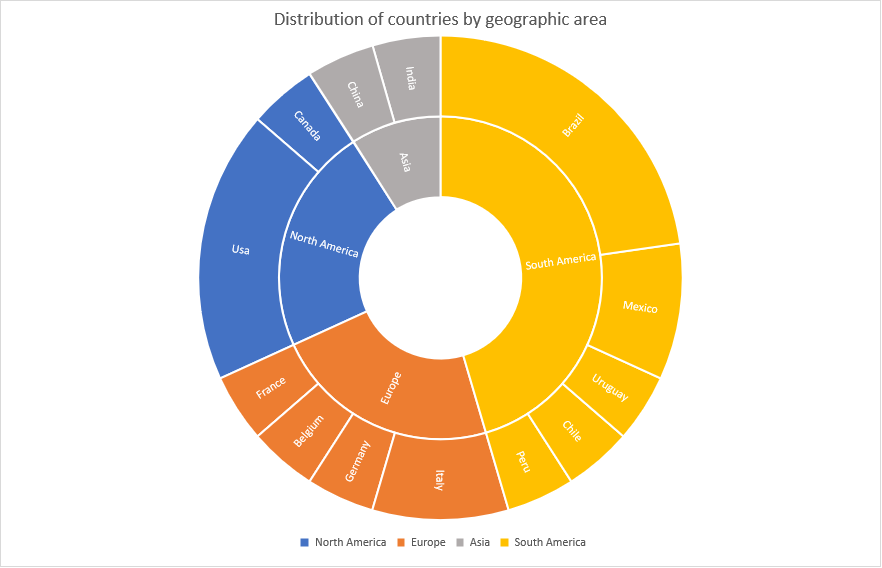


**Supplementary Figure 2** Representation of geographic areas of the studies included in the analysis for cancer diagnosis


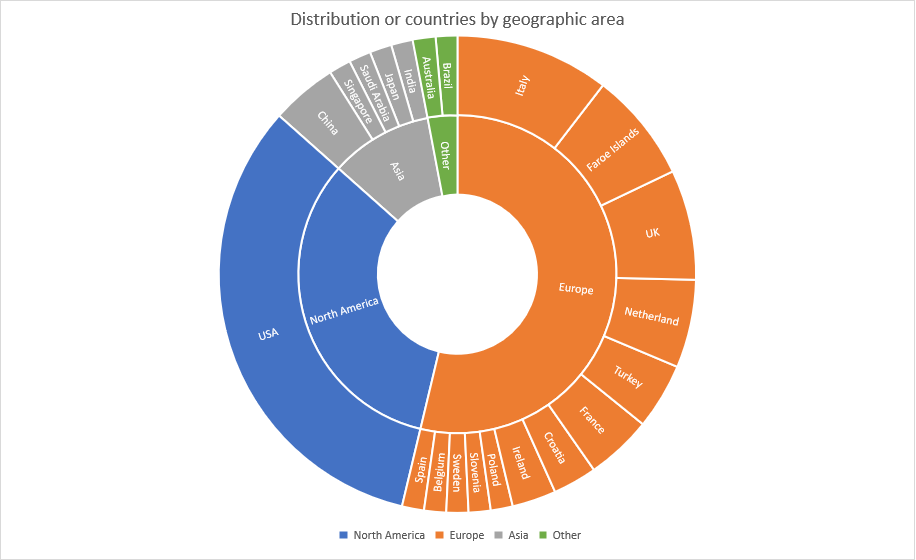


**Supplementary Figure 3** Representation of the online publication date of the studies included in the analysis for cancer diagnostic tests by quarter.

**Supplementary Figure 4** Representation of the online publication date of the studies included in the analysis for cancer diagnosis by quarter.

**References**

1. Eijkelboom AH, de Munck L, Vrancken Peeters MJTFD, Broeders MJM, Strobbe LJA, Bos MEMM, et al. Impact of the COVID-19 pandemic on diagnosis, stage, and initial treatment of breast cancer in the Netherlands: a population-based study. J Hematol OncolJ Hematol Oncol. 2021 Apr 17;14(1):64.

2. Karacin C, Acar R, Bal O, Eren T, Sendur MAN, Acikgoz Y, et al. “Swords and Shields” against COVID-19 for patients with cancer at “clean” and “pandemic” hospitals: are we ready for the second wave? Support Care Cancer Off J Multinatl Assoc Support Care Cancer. 2021 Aug;29(8):4587–93.

3. Chiaravalli S, Ferrari A, Sironi G, Gattuso G, Bergamaschi L, Puma N, et al. A collateral effect of the COVID-19 pandemic: Delayed diagnosis in pediatric solid tumors. Pediatr Blood Cancer. 2020 Oct;67(10):e28640.

4. Ns Z, Jl W, Tj O, Qs W, Xo S, Sa D, et al. A retrospective approach to evaluating potential adverse outcomes associated with delay of procedures for cardiovascular and cancer-related diagnoses in the context of COVID-19. J Biomed Inform [Internet]. 2021 Jan [cited 2022 Jun 21];113. Available from: https://pubmed.ncbi.nlm.nih.gov/33309899/

5. Barruscotti S, Giorgini C, Brazzelli V, Vassallo C, Michelerio A, Klersy C, et al. A significant reduction in the diagnosis of melanoma during the COVID-19 lockdown in a third-level center in the Northern Italy. Dermatol Ther. 2020 Nov;33(6):e14074.

6. Kristiansen MF, Petersen MS, Strøm M. Cancer diagnosed during the COVID-19 pandemic in the Faroe Islands. Acta Oncol Stockh Swed. 2021 Jul;60(7):856–8.

7. Marques NP, Silveira DMM, Marques NCT, Martelli DRB, Oliveira EA, Martelli-Júnior H. Cancer diagnosis in Brazil in the COVID-19 era. Semin Oncol. 2021 Apr;48(2):156–9.

8. De Vincentiis L, Carr RA, Mariani MP, Ferrara G. Cancer diagnostic rates during the 2020 “lockdown”, due to COVID-19 pandemic, compared with the 2018-2019: an audit study from cellular pathology. J Clin Pathol. 2021 Mar;74(3):187–9.

9. Z B, M P, Al S, Sr L, Tk C, Qd T. Cancer Screening Tests and Cancer Diagnoses During the COVID-19 Pandemic. JAMA Oncol [Internet]. 2021 Jan 3 [cited 2022 Jun 21];7(3). Available from: https://pubmed.ncbi.nlm.nih.gov/33443549/

10. Buscarini E, Benedetti A, Monica F, Pasquale L, Buttitta F, Cameletti M, et al. Changes in digestive cancer diagnosis during the SARS-CoV-2 pandemic in Italy: A nationwide survey. Dig Liver Dis Off J Ital Soc Gastroenterol Ital Assoc Study Liver. 2021 Jun;53(6):682–8.

11. Sprague BL, Lowry KP, Miglioretti DL, Alsheik N, Bowles EJA, Tosteson ANA, et al. Changes in Mammography Use by Women’s Characteristics During the First 5 Months of the COVID-19 Pandemic. J Natl Cancer Inst. 2021 Sep 4;113(9):1161–7.

12. Kaufman HW, Chen Z, Niles JK, Fesko YA. Changes in Newly Identified Cancer Among US Patients From Before COVID-19 Through the First Full Year of the Pandemic. JAMA Netw Open. 2021 Aug 2;4(8):e2125681.

13. Pucci R, Cassoni A, Battisti A, Valentini V. Covid-19 pandemic and head and neck cancers, what should we expect? Oral Oncol. 2021 Sep;120:105263.

14. E V, Mp B, Z M, M A, V B, Č T, et al. COVID-19 Pandemic Effects on Breast Cancer Diagnosis in Croatia: A Population- and Registry-Based Study. The oncologist [Internet]. 2021 Jul [cited 2022 Jun 21];26(7). Available from: https://pubmed.ncbi.nlm.nih.gov/33856084/

15. Maluchnik M, Podwójcic K, Więckowska B. Decreasing access to cancer diagnosis and treatment during the COVID-19 pandemic in Poland. Acta Oncol Stockh Swed. 2021 Jan;60(1):28–31.

16. Koca B, Yildirim M. Delay in breast cancer diagnosis and its clinical consequences during the coronavirus disease pandemic. J Surg Oncol. 2021 Sep;124(3):261–7.

17. Offenbacher R, Knoll MA, Loeb DM. Delayed presentations of pediatric solid tumors at a tertiary care hospital in the Bronx due to COVID-19. Pediatr Blood Cancer. 2021 Feb;68(2):e28615.

18. Vardhanabhuti V, Ng KS. Differential Impact of COVID-19 on Cancer Diagnostic Services Based on Body Regions: A Public Facility-Based Study in Hong Kong. Int J Radiat Oncol Biol Phys. 2021 Oct 1;111(2):331–6.

19. Song H, Bergman A, Chen AT, Ellis D, David G, Friedman AB, et al. Disruptions in preventive care: Mammograms during the COVID-19 pandemic. Health Serv Res. 2021 Feb;56(1):95–101.

20. London JW, Fazio-Eynullayeva E, Palchuk MB, Sankey P, McNair C. Effects of the COVID-19 Pandemic on Cancer-Related Patient Encounters. JCO Clin Cancer Inform. 2020 Jul;4:657–65.

21. Md W, P G, B H, Tm J, Aj K. Emergency presentations of head and neck cancer: Our experience in the wake of the COVID-19 pandemic. Clin Otolaryngol Off J ENT-UK Off J Neth Soc Oto-Rhino-Laryngol Cervico-Facial Surg [Internet]. 2021 Nov [cited 2022 Jun 21];46(6). Available from: https://pubmed.ncbi.nlm.nih.gov/34097807/

22. Suh-Burgmann EJ, Alavi M, Schmittdiel J. Endometrial Cancer Detection During the Coronavirus Disease 2019 (COVID-19) Pandemic. Obstet Gynecol. 2020 Oct;136(4):842–3.

23. Kuzuu K, Misawa N, Ashikari K, Kessoku T, Kato S, Hosono K, et al. Gastrointestinal Cancer Stage at Diagnosis Before and During the COVID-19 Pandemic in Japan. JAMA Netw Open. 2021 Sep 1;4(9):e2126334.

24. Vissio E, Falco EC, Collemi G, Borella F, Papotti M, Scarmozzino A, et al. Impact of COVID-19 lockdown measures on oncological surgical activity: Analysis of the surgical pathology caseload of a tertiary referral hospital in Northwestern Italy. J Surg Oncol. 2021 Jan;123(1):24–31.

25. Ahmad N, Essa MF, Sudairy R. Impact of Covid19 on a tertiary care pediatric oncology and stem cell transplant unit in Riyadh, Saudi Arabia. Pediatr Blood Cancer. 2020 Sep;67(9):e28560.

26. Zadnik V, Mihor A, Tomsic S, Zagar T, Bric N, Lokar K, et al. Impact of COVID-19 on cancer diagnosis and management in Slovenia - preliminary results. Radiol Oncol. 2020 Jul 29;54(3):329–34.

27. Wang H, Elsheikh M, Gilmour K, Cohen V, Sagoo MS, Damato B, et al. Impact of COVID-19 pandemic on eye cancer care in United Kingdom. Br J Cancer. 2021 Apr;124(8):1357–60.

28. Khan A, Bilal M, Morrow V, Cooper G, Thakkar S, Singh S. Impact of the Coronavirus Disease 2019 Pandemic on Gastrointestinal Procedures and Cancers in the United States: A Multicenter Research Network Study. Gastroenterology. 2021 Jun;160(7):2602-2604.e5.

29. Rutter MD, Brookes M, Lee TJ, Rogers P, Sharp L. Impact of the COVID-19 pandemic on UK endoscopic activity and cancer detection: a National Endoscopy Database Analysis. Gut. 2021 Mar;70(3):537–43.

30. Eijkelboom AH, de Munck L, Lobbes MBI, van Gils CH, Wesseling J, Westenend PJ, et al. Impact of the suspension and restart of the Dutch breast cancer screening program on breast cancer incidence and stage during the COVID-19 pandemic. Prev Med. 2021 Oct;151:106602.

31. Fernández Canedo MI, de Troya Martín M, Rivas Ruíz F. Impact of the SARS-CoV-2 pandemic on the early diagnosis of melanoma. Med Clin Engl Ed. 2021 Apr 9;156(7):356–7.

32. Lui TKL, Leung K, Guo CG, Tsui VWM, Wu JT, Leung WK. Impacts of the Coronavirus 2019 Pandemic on Gastrointestinal Endoscopy Volume and Diagnosis of Gastric and Colorectal Cancers: A Population-Based Study. Gastroenterology. 2020 Sep;159(3):1164-1166.e3.

33. Hamilton AC, Donnelly DW, Loughrey MB, Turkington RC, Fox C, Fitzpatrick D, et al. Inequalities in the decline and recovery of pathological cancer diagnoses during the first six months of the COVID-19 pandemic: a population-based study. Br J Cancer. 2021 Sep;125(6):798–805.

34. Ferro A, Cristofolini P, Garcia-Etienne CA, Caffo O, Pellegrini M, Fantò C, et al. Learning from organisational changes in the management of breast cancer patients during the COVID-19 pandemic: Preparing for a second wave at a breast unit in northern Italy. Int J Health Plann Manage. 2021 Jul;36(4):1030–7.

35. Penel N, Hammoudi A, Marliot G, De Courreges A, Cucchi M, Mirabel X, et al. Major impact of COVID-19 national containment on activities in the French northern comprehensive cancer center. Med Oncol Northwood Lond Engl. 2021 Feb 17;38(3):28.

36. Rasschaert M, Vanclooster P, Depauw L, Mertens T, Roelant E, Coenen E, et al. Meeting the Challenges in Cancer Care Management During the SARS-Cov-2 Pandemic: A Retrospective Analysis. Cancer Control J Moffitt Cancer Cent. 2021 Dec;28:10732748211045276.

37. Chiu HM, Su CW, Hsu WF, Jen GHH, Hsu CY, Chen SLS, et al. Mitigating the impact of COVID-19 on colorectal cancer screening: Organized service screening perspectives from the Asia-Pacific region. Prev Med. 2021 Oct;151:106622.

38. Kempf E, Lamé G, Layese R, Priou S, Chatellier G, Chaieb H, et al. New cancer cases at the time of SARS-Cov2 pandemic and related public health policies: A persistent and concerning decrease long after the end of the national lockdown. Eur J Cancer Oxf Engl 1990. 2021 Jun;150:260–7.

39. Trehan A, Jain R, Bansal D. Oncology care in a lower middle-income country during the COVID-19 pandemic. Pediatr Blood Cancer. 2020 Aug;67(8):e28438.

40. Hw K, Z C, Jk N, J R, Y F. Patterns of Prostate-Specific Antigen Testing and Prostate Biopsies During the COVID-19 Pandemic. JCO Clin Cancer Inform [Internet]. 2021 Sep [cited 2022 Jun 21];5. Available from: https://pubmed.ncbi.nlm.nih.gov/34648367/

41. Nyante SJ, Benefield TS, Kuzmiak CM, Earnhardt K, Pritchard M, Henderson LM. Population-level impact of coronavirus disease 2019 on breast cancer screening and diagnostic procedures. Cancer. 2021 Jun 15;127(12):2111–21.

42. G F, F S, J S, S C, If L, J A, et al. Prostate cancer diagnosis, staging, and treatment in Sweden during the first phase of the COVID-19 pandemic. Scand J Urol [Internet]. 2021 Jun [cited 2022 Jun 21];55(3). Available from: https://pubmed.ncbi.nlm.nih.gov/33913376/

43. Labaki C, Bakouny Z, Schmidt A, Lipsitz SR, Rebbeck TR, Trinh QD, et al. Recovery of cancer screening tests and possible associated disparities after the first peak of the COVID-19 pandemic. Cancer Cell. 2021 Aug 9;39(8):1042–4.

44. Andrew TW, Alrawi M, Lovat P. Reduction in skin cancer diagnoses in the UK during the COVID-19 pandemic. Clin Exp Dermatol. 2021 Jan;46(1):145–6.

45. A M, M Y, Vi A, T Z, Jm H, Bj D, et al. Referral pattern for urologic malignancies before and during the COVID-19 pandemic. Urol Oncol [Internet]. 2021 May [cited 2022 Jun 21];39(5). Available from: https://pubmed.ncbi.nlm.nih.gov/33308974/

46. Roseleur J, Gonzalez-Chica DA, Emery J, Stocks NP. Skin checks and skin cancer diagnosis in Australian general practice before and during the COVID-19 pandemic, 2011-2020. Br J Dermatol. 2021 Oct;185(4):853–5.

47. Kiong KL, Diaz EM, Gross ND, Diaz EM, Hanna EY. The impact of COVID-19 on head and neck cancer diagnosis and disease extent. Head Neck. 2021 Jun;43(6):1890–7.

48. Turkington RC, Lavery A, Donnelly D, Cairnduff V, McManus DT, Coleman HG. The Impact of the COVID-19 Pandemic on Barrett’s Esophagus and Esophagogastric Cancer. Gastroenterology. 2021 May;160(6):2169-2171.e1.

49. Tl K, L de J, Pha W, I S, P OT, Me van L, et al. The national FIT-based colorectal cancer screening program in the Netherlands during the COVID-19 pandemic. Prev Med [Internet]. 2021 Oct [cited 2022 Jun 21];151. Available from: https://pubmed.ncbi.nlm.nih.gov/34217421/

50. Marson JW, Maner BS, Harding TP, Meisenheimer J, Solomon JA, Leavitt M, et al. The magnitude of COVID-19’s effect on the timely management of melanoma and nonmelanoma skin cancers. J Am Acad Dermatol. 2021 Apr;84(4):1100–3.

51. Asai Y, Nguyen P, Hanna TP. Impact of the COVID-19 pandemic on skin cancer diagnosis: A population-based study. PloS One. 2021;16(3):e0248492.

52. Palladino R, Migliatico I, Sgariglia R, Nacchio M, Iaccarino A, Malapelle U, et al. Thyroid fine-needle aspiration trends before, during, and after the lockdown: what we have learned so far from the COVID-19 pandemic. Endocrine. 2021 Jan;71(1):20–5.

53. de Pelsemaeker MC, Guiot Y, Vanderveken J, Galant C, Van Bockstal MR. The Impact of the COVID-19 Pandemic and the Associated Belgian Governmental Measures on Cancer Screening, Surgical Pathology and Cytopathology. Pathobiol J Immunopathol Mol Cell Biol. 2021;88(1):46–55.

54. Ranganathan P, Sengar M, Chinnaswamy G, Agrawal G, Arumugham R, Bhatt R, et al. Impact of COVID-19 on cancer care in India: a cohort study. Lancet Oncol. 2021 Jul;22(7):970–6.

55. Patt D, Gordan L, Diaz M, Okon T, Grady L, Harmison M, et al. Impact of COVID-19 on Cancer Care: How the Pandemic Is Delaying Cancer Diagnosis and Treatment for American Seniors. JCO Clin Cancer Inform. 2020 Nov;4:1059–71.

56. Zattra O, Fraga A, Lu N, Gee MS, Liu RW, Lev MH, et al. Trends in cancer imaging by indication, care setting, and hospital type during the COVID-19 pandemic and recovery at four hospitals in Massachusetts. Cancer Med. 2021 Sep;10(18):6327–35.

57. O M, N J, J G, Mj W, M R, L R, et al. The impact of the COVID-19 pandemic on the Ontario Cervical Screening Program, colposcopy and treatment services in Ontario, Canada: a population-based study. BJOG Int J Obstet Gynaecol [Internet]. 2021 Aug [cited 2022 Jun 21];128(9). Available from: https://pubmed.ncbi.nlm.nih.gov/33982870/

58. Vázquez Rosas T, Cazap E, Delgado L, Ismael J, Bejarano S, Castro C, et al. Social Distancing and Economic Crisis During COVID-19 Pandemic Reduced Cancer Control in Latin America and Will Result in Increased Late-Stage Diagnoses and Expense. JCO Glob Oncol. 2021 May;7:694–703.

59. Fonseca GA, Normando PG, Loureiro LVM, Rodrigues REF, Oliveira VA, Melo MDT, et al. Reduction in the Number of Procedures and Hospitalizations and Increase in Cancer Mortality During the COVID-19 Pandemic in Brazil. JCO Glob Oncol. 2021 Jan;7:4–9.

60. Brugel M, Carlier C, Essner C, Debreuve-Theresette A, Beck MF, Merrouche Y, et al. Dramatic Changes in Oncology Care Pathways During the COVID-19 Pandemic: The French ONCOCARE-COV Study. The Oncologist. 2021 Feb;26(2):e338–41.

61. Stang A, Kühling L, Khil L, Kajüter H, Schützendübel A, Mattauch V. Drop in Cancer Reporting by Pathologists in North Rhine-Westphalia, Germany, During the COVID-19 Lockdown. Dtsch Arzteblatt Int. 2020 Dec 21;117(51–52):886–7.
